# Supplementary material for: Interplay and cooperation between SREBF1 and master transcription factors regulate lipid metabolism and tumor-promoting pathways in squamous cancer
Source: Nat Commun. 2021 Jul 16;12:4362. doi: 10.1038/s41467-021-24656-x (PMC8285542; doi:10.1038/s41467-021-24656-x)
Supplement: Supplementary file 1 — Supplementary Information [file 41467_2021_24656_MOESM1_ESM.pdf]

Supplementary Figure 1

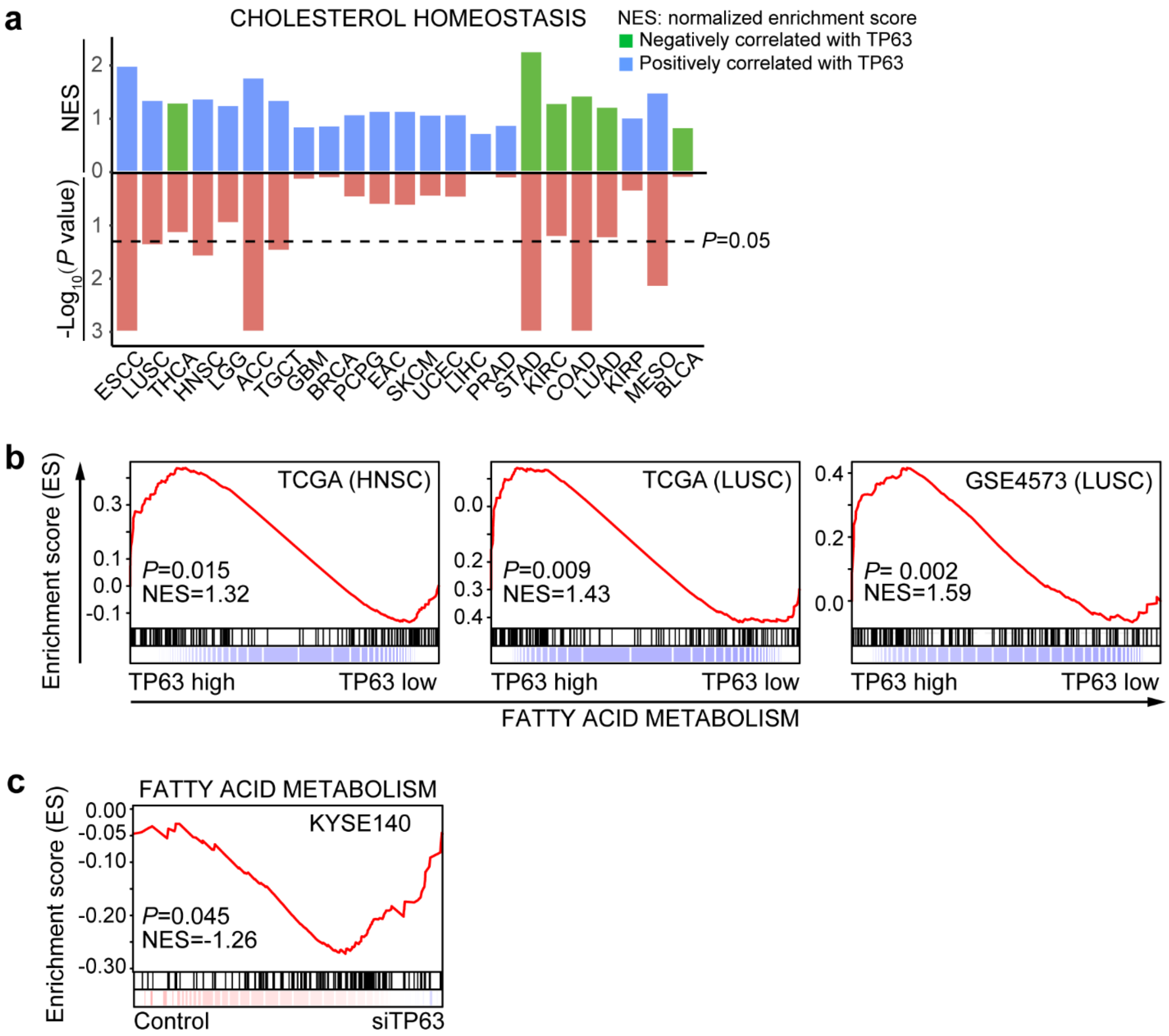

Supplementary Figure 1

(a) Bar plots showing the NES (normalized enrichment score, upper) and *P* value (lower) from GSEA results of cholesterol homeostasis pathway in TP63-high samples from 23 types of cancers from TCGA.

(b) Individual GSEA plots of fatty-acid metabolism pathway in the TCGA cohort of HNSC samples and two independent cohorts of LUSC samples (TCGA and GSE4573).

(c) GSEA plot of fatty-acid metabolism pathway in RNA-Seq data upon silencing of TP63 in KYSE140 cells. NES, normalized enrichment score. *P* values in panel (a-c) were adjusted for multiple comparisons.

Supplementary Figure 2

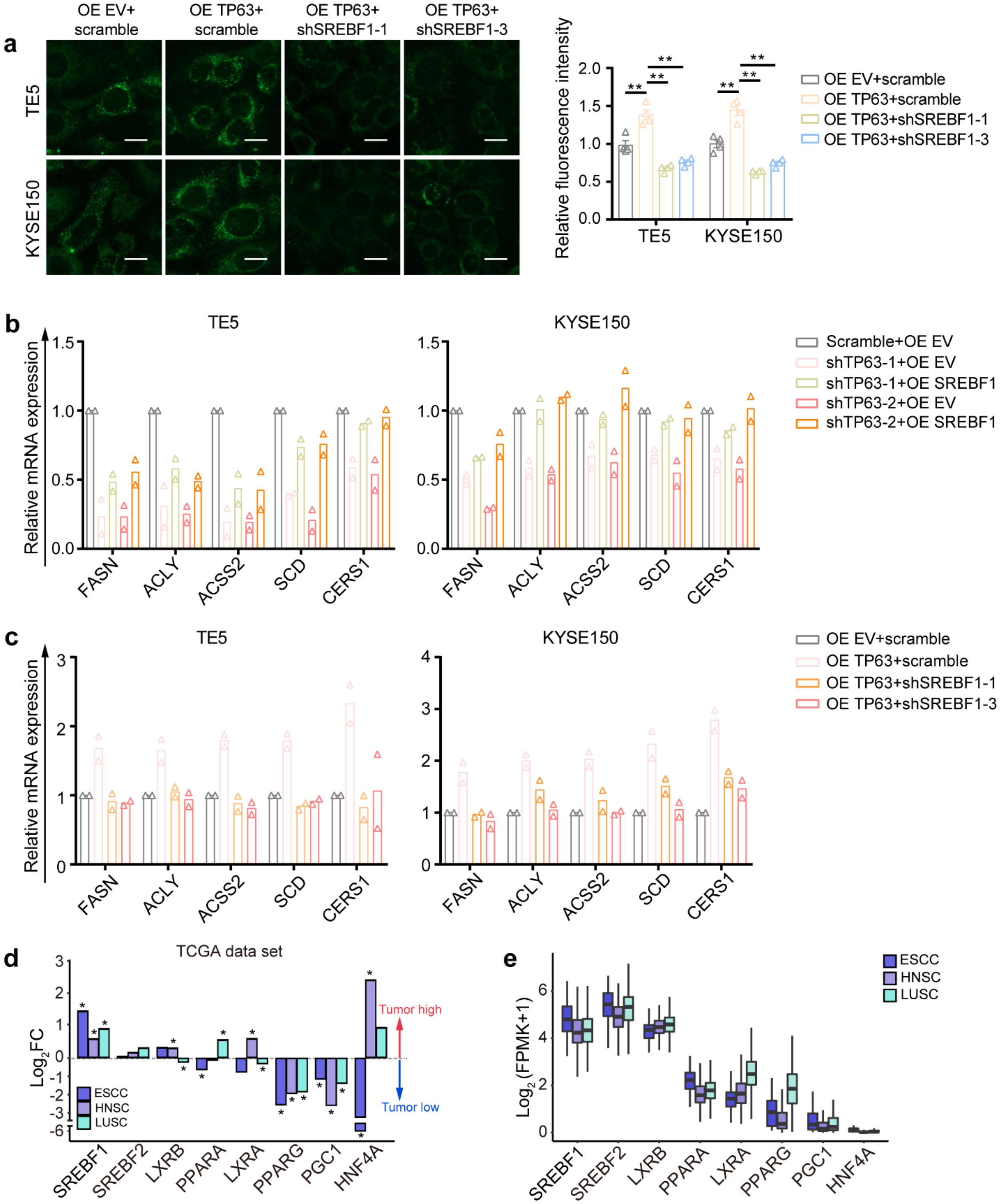

Supplementary Figure 2

(a) TE5 and KYSE150 cells were transfected with plasmids encoding either empty vector (OE EV) or TP63 (OE TP63) for 24 hours, followed by the transfection of shRNA plasmids targeting either SREBF1 or scramble for another 48 hours. Lipid droplet was stained and analyzed with confocal microscopy. Fluorescence intensity was quantified in each group and plotted as fold-changes relative to the control group of OE EV+scramble. Mean±SEM are shown; n=4 (the number of microscopic vision); **, $P<0.01$, P values were determined by a two-sided t-test. Scale bar, 50 μm.
(b) TE5 and KYSE150 cells were transfected with shRNA plasmids targeting either TP63 or scramble for 24 hours, followed by the transfection of plasmids encoding either empty vector (OE EV) or SREBF1 (OE SREBF1) for another 48 hours. The samples were then subjected to qRT-PCR experiments. Barplots displaying the fold changes relative to control. Mean values are shown; n=2 (biological replicates).
(c) TE5 and KYSE150 cells were transfected with plasmids encoding either empty vector (OE EV) or TP63 (OE TP63) for 24 hours, followed by the transfection of shRNA plasmids targeting either SREBF1 or scramble for another 48 hours. The samples were then subjected to qRT-PCR experiments. Barplots displaying the fold changes relative to control. Mean values are shown; n=2 (biological replicates).
(d) Fold changes of mRNA levels of indicated genes in SCC tumors compared with nonmalignant samples from the TCGA cohorts. Limma R package was used to calculate the P values based on the tumor and the corresponding normal samples in ESCC, HNSC (non-HPV+) and LUSC. *, unadjusted $P<0.05$.
(e) Log2(FPKM) values of mRNAs of indicated genes in SCC tumors from TCGA cohorts. The box plots indicate the median (middle line), 25th, 75th percentile (box) and 5th and 95th percentile (whiskers); The numbers of samples are 81 (ESCC), 436 (HNSC) and 501 (LUSC).

Supplementary Figure 3

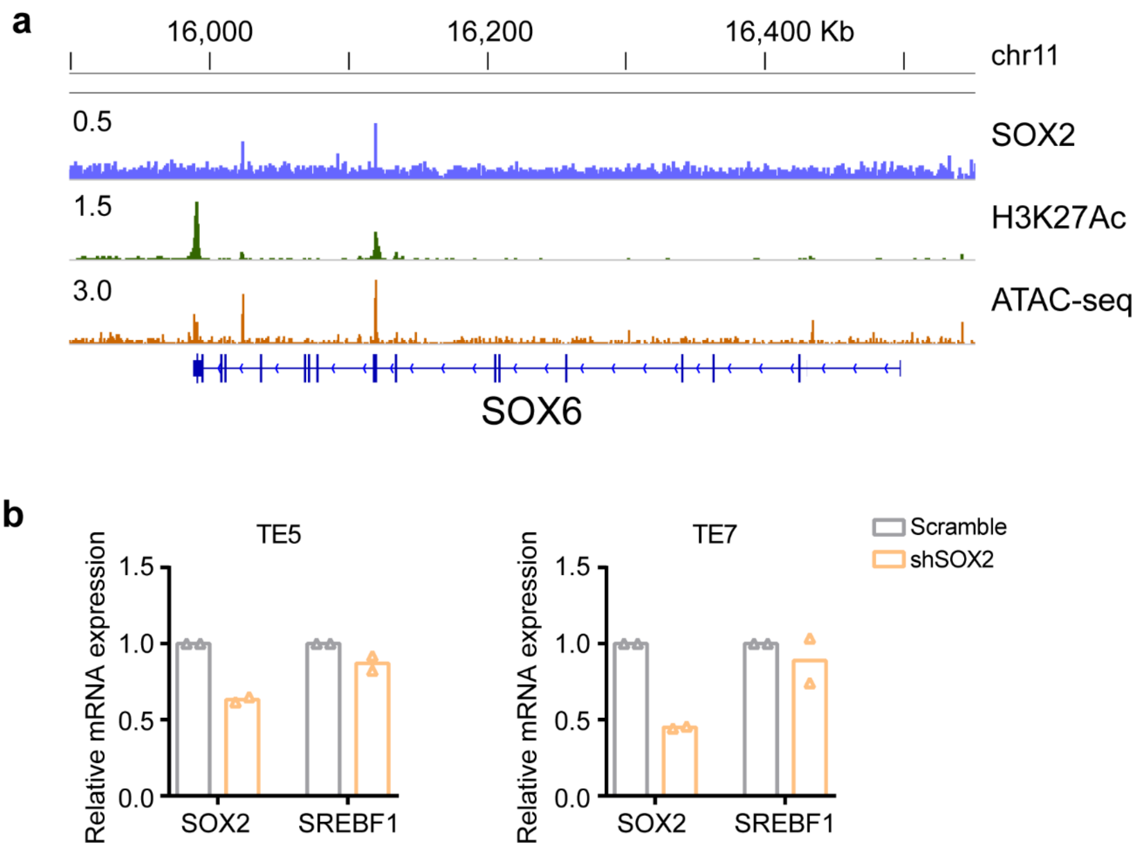

Supplementary Figure 3

(a) IGV tracks of ChIP-Seq of SOX2 and H3K27Ac as well as ATAC-Seq at *SOX6* locus in TE5 cells.

(b) qRT-PCR results of the mRNA expression of SOX2 and SREBF1 upon the silencing of SOX2 in TE5 and TE7 cells. Mean values are shown; n=2 (biological replicates).

Supplementary Figure 4

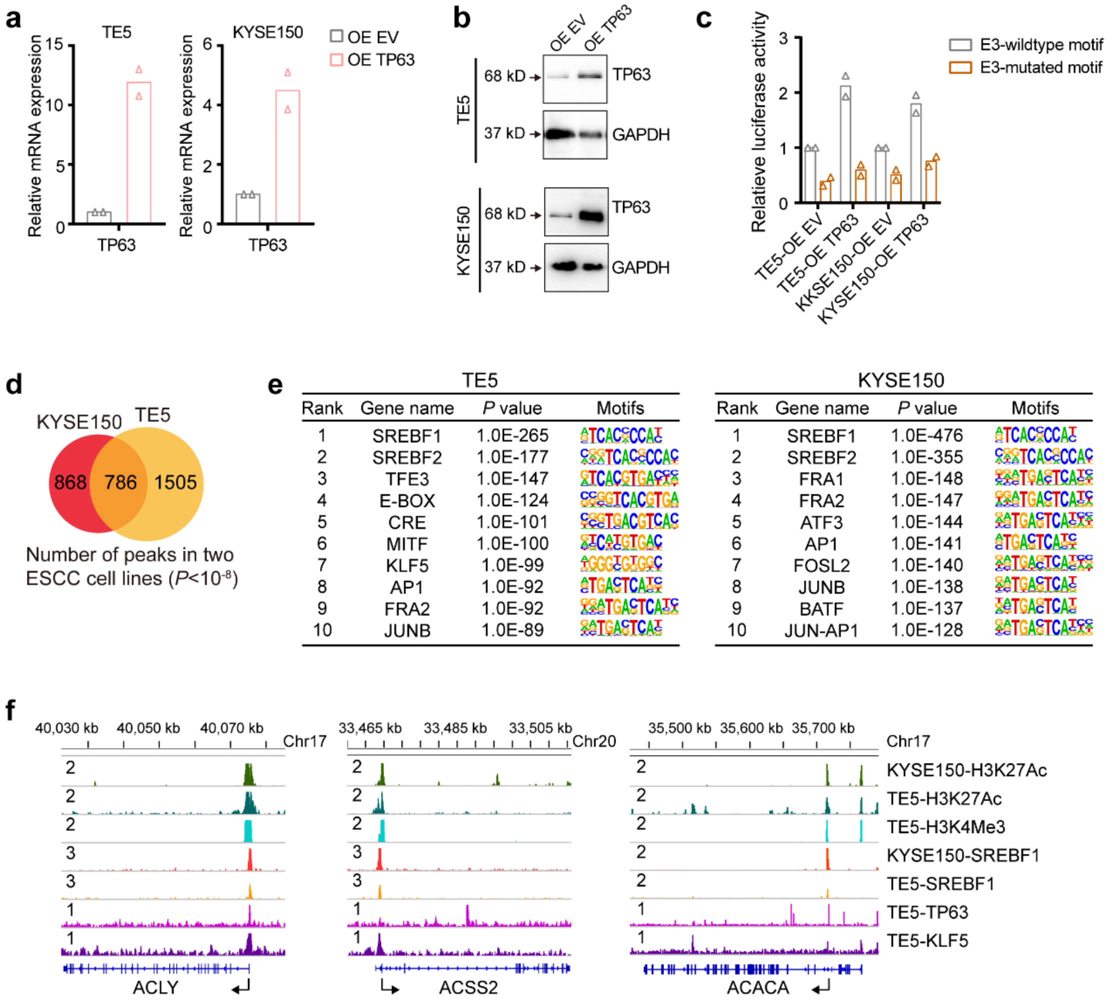

Supplementary Figure 4

(a) qRT-PCR and **(b)** Western blotting analyses of the overexpression efficiency 48 hours after the transfection with either empty vector (OE EV) or TP63 (OE TP63) in ESCC cell lines. Mean values are shown; n=2 (biological replicates).

(c) TE5 and KYSE150 cells were transfected with either empty vector (OE EV) or TP63 (OE TP63) for 24 hours, and then co-transfected with both the renilla plasmid and pGL3-promoter plasmid containing either wild-type or mutant E3 enhancer for another 48 hours. The relative transcriptional reporter activity was analyzed by dual luciferase reporter gene assay. Mean values are shown; n=2 (biological replicates).

(d) SREBF1 binding peaks strongly overlapped in TE5 and KYSE150 cell lines. Simple Random Sampling with Replacement was used and the *P* value is the proportion that the number of randomly overlapping peaks is greater than the number of truly overlapping peaks (786). The number of random is 108, and the unadjusted *P*=0.

(e) Top 10 most significantly enriched motifs in SREBF1 ChIP-Seq peaks in TE5 and KYSE150 cells.

(f) IGV tracks of ChIP-Seq profiles of indicated factors at the loci of canonical SREBF1 target genes in TE5 and KYSE150 cell lines.

Supplementary Figure 5

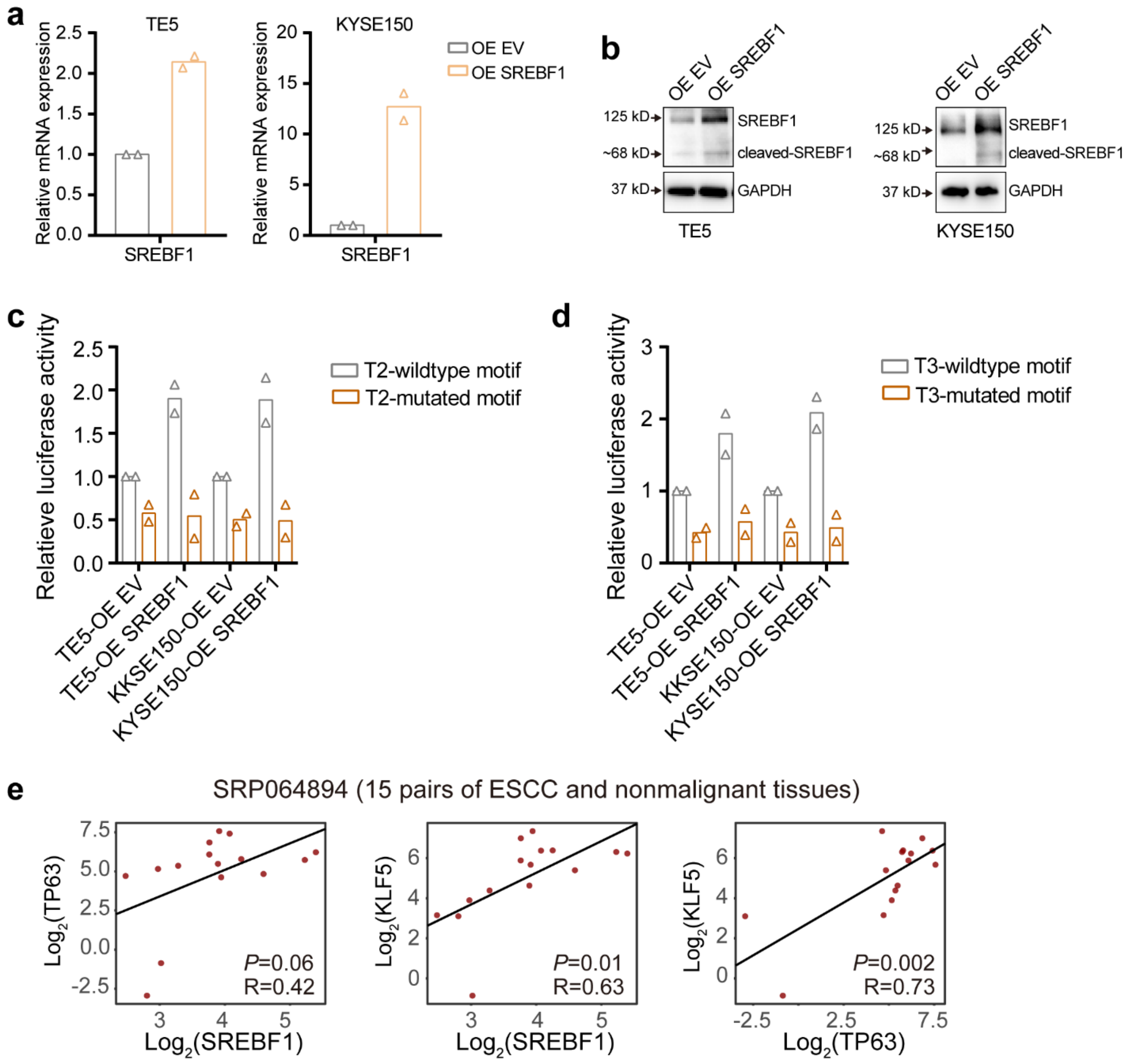

Supplementary Figure 5

(a) qRT-PCR and **(b)** Western blotting analyses of the overexpression efficiency 48 hours after the transfection with either empty vector (OE EV) or SREBF1 (OE SREBF1) in ESCC cell lines. Mean values are shown; n=2 (biological replicates). The western blotting experiments were performed in three biologically independent replicates.

(c) TE5 and **(d)** KYSE150 cells were transfected with either empty vector (OE EV) or SREBF1 (OE SREBF1) for 24 hours, and then co-transfected with both the renilla plasmid and pGL3-promoter plasmid containing either wild-type or mutant T2/T3 enhancers for another 48 hours.

The relative transcriptional reporter activity was analyzed by dual luciferase reporter gene assay. Mean values are shown; n=2 (biological replicates).

(e) Pearson correlation coefficient between the mRNA levels of SREBF1, TP63 and KLF5 in the SRP064894 dataset containing 15 pairs of ESCC and nonmalignant esophageal epithelium samples. Unajusted *P* values are shown.

Supplementary Figure 6

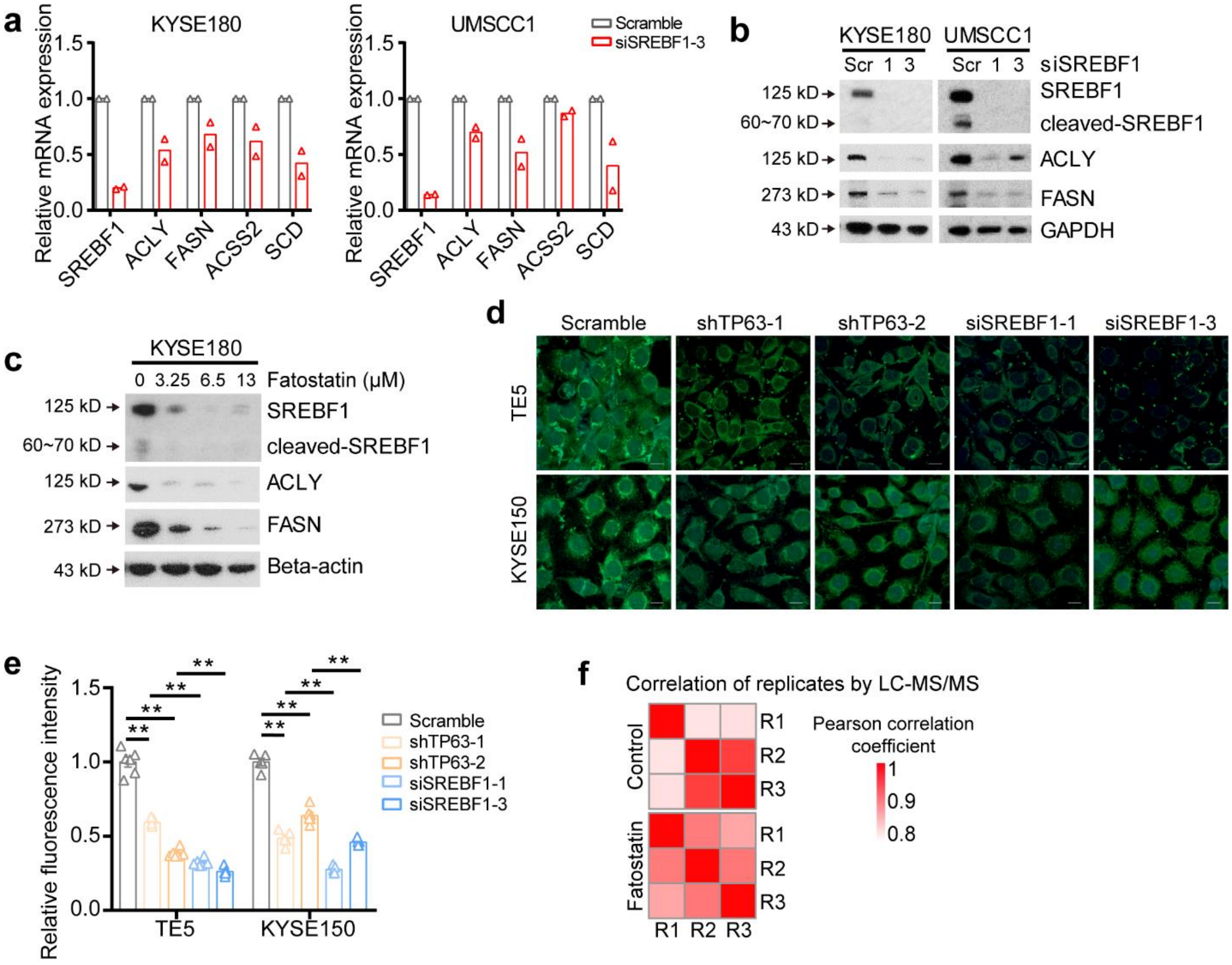

Supplementary Figure 6

(a) qRT-PCR measuring mRNA levels of central enzymes for fatty-acid synthesis upon knockdown of SREBF1 in KYSE180 and UMSCC1 cells. Mean values are shown; n=2 (biological replicates).

(b) Western blotting analyses showing protein levels of central enzymes for fatty-acid synthesis upon knockdown of SREBF1. The western blotting experiments were performed in three biologically independent replicates.

(c) Western blotting analyses showing protein levels of central enzymes for fatty-acid synthesis upon treatment with Fatostatin. The western blotting experiments were performed in three biologically independent replicates.

(d) TP63 and SREBF1 were silenced individually in TE5 and KYSE150 cells and lipid droplet staining was analyzed with confocal microscopy. Scale bar, 50 μm.

(e) Fluorescence intensity was quantified and shown in the bar plots. Mean ± SEM are shown; n=5 except for the groups of TE5-scramble and TE5-siSREBF1-1 (n=6), as the number of microscopic vision; **, $P<0.01$, P values were determined by a two-sided t-test.

(f) Heatmap showing correlation between triplicates of both control and experimental samples by LC-MS/MS-based lipidomics after treatment with Fatostatin in KYSE510 cells.

Supplementary Figure 7

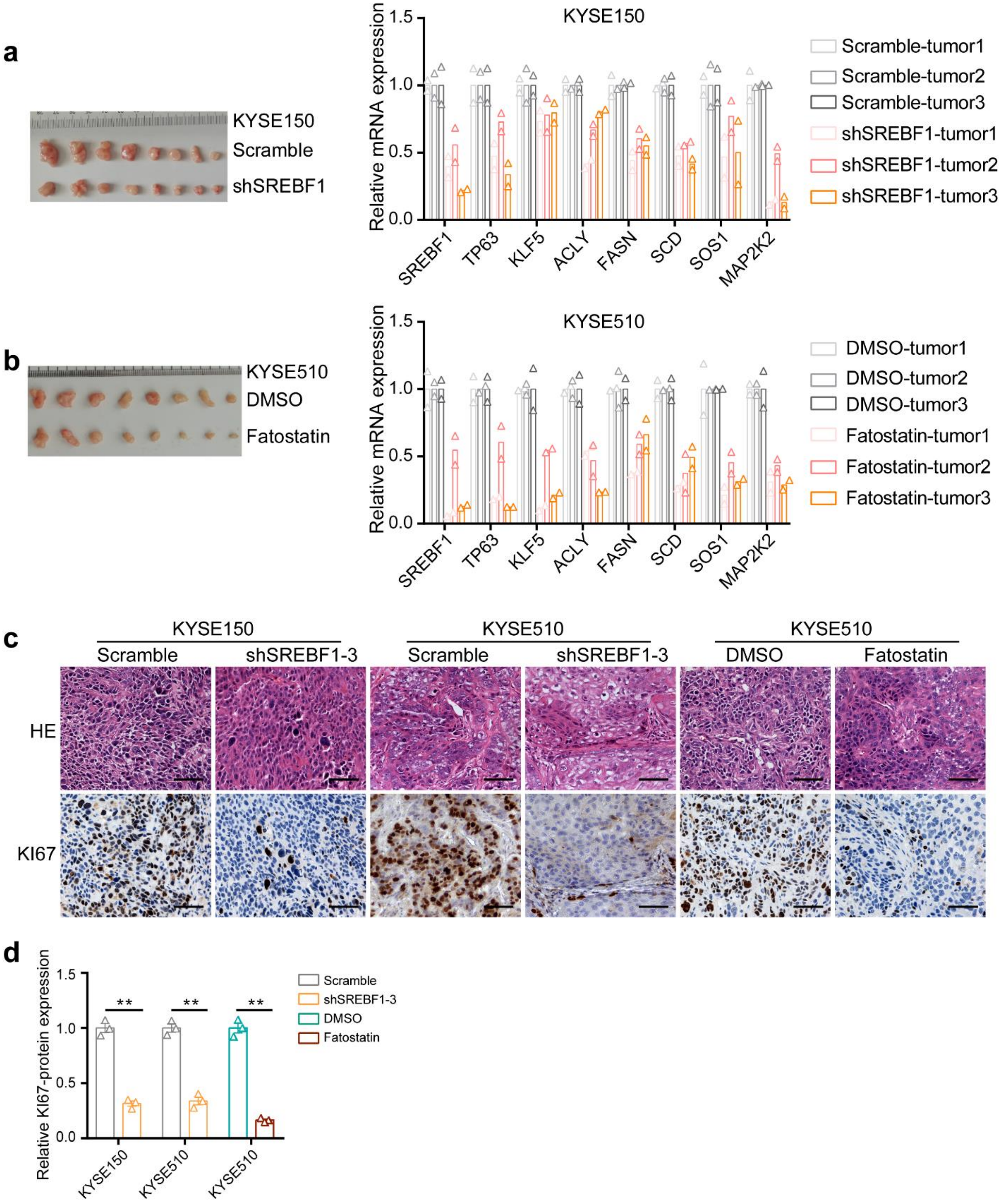

Supplementary Figure 7

(a) and **(b)** mRNA expression of indicated genes in the xenograft tumors quantified by qRT-PCR. Mean values are shown; n=2 (biological replicates).

(c) Paraffin-embedded xenograft samples were stained using hematoxylin-eosin (HE) and immunohistochemistry (IHC) for KI67. Original magnification, 400 × ; scale bar, 50 μm.

(d) Protein expression of KI67 in **(c)** was quantified in each group and plotted as fold-changes relative to the control group. Mean ± SEM; n=3 (the number of microscopic vision); **, $P<0.01$, P values were determined by a two-sided t-test.

Supplementary Figure 8

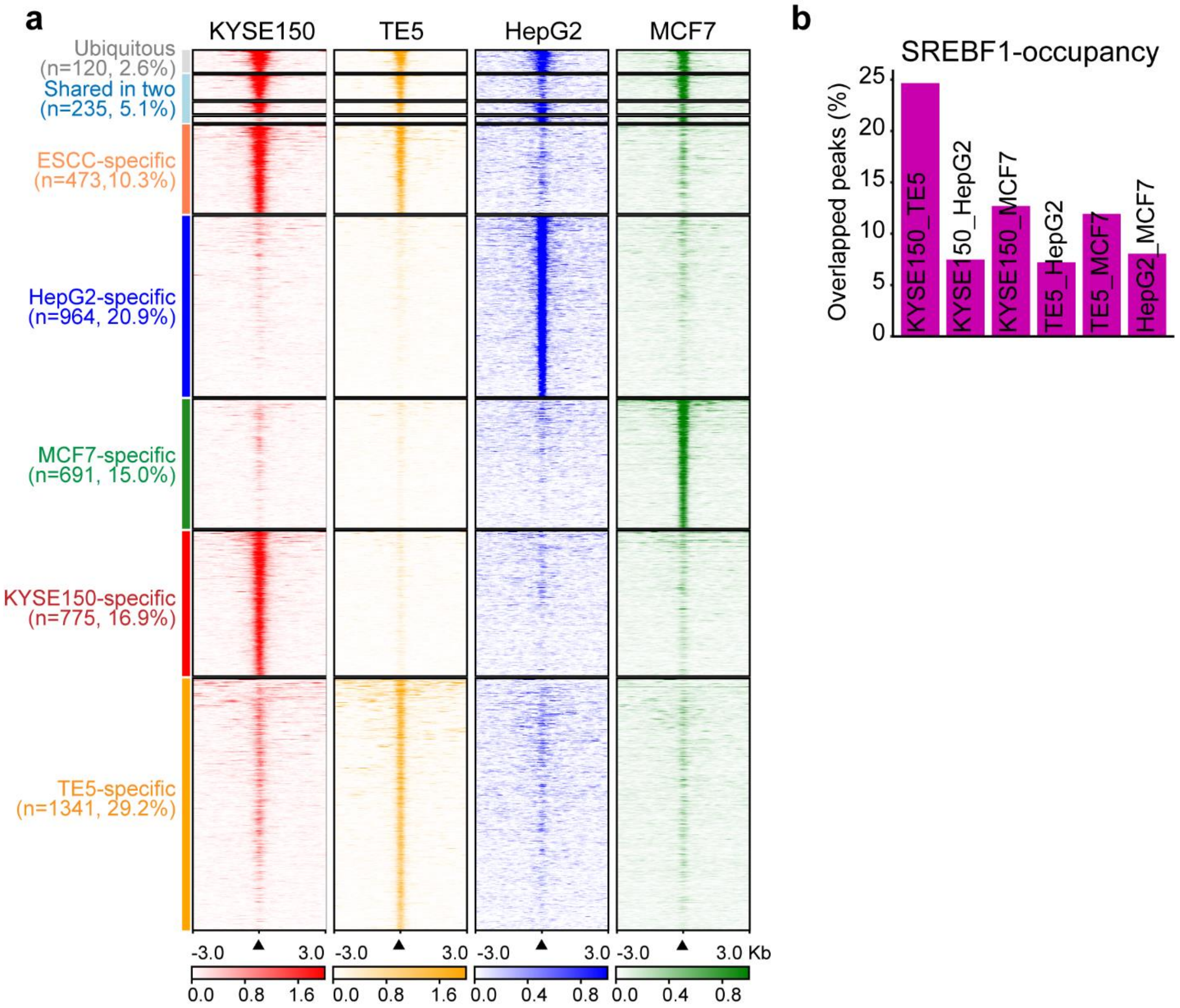

Supplementary Figure 8

(a) Heatmaps of ChIP-Seq signals at SREBF1-peak regions (±3 Kb of peak center), rank ordered by the intensity of peaks based on reads per million mapped reads (RPM). Lines, peaks; color scale of peak intensity is shown at the bottom.

(**b)** The ratio of overlapped SREBF1-binding peaks overall all peaks between any two cell lines.

Supplementary Figure 9

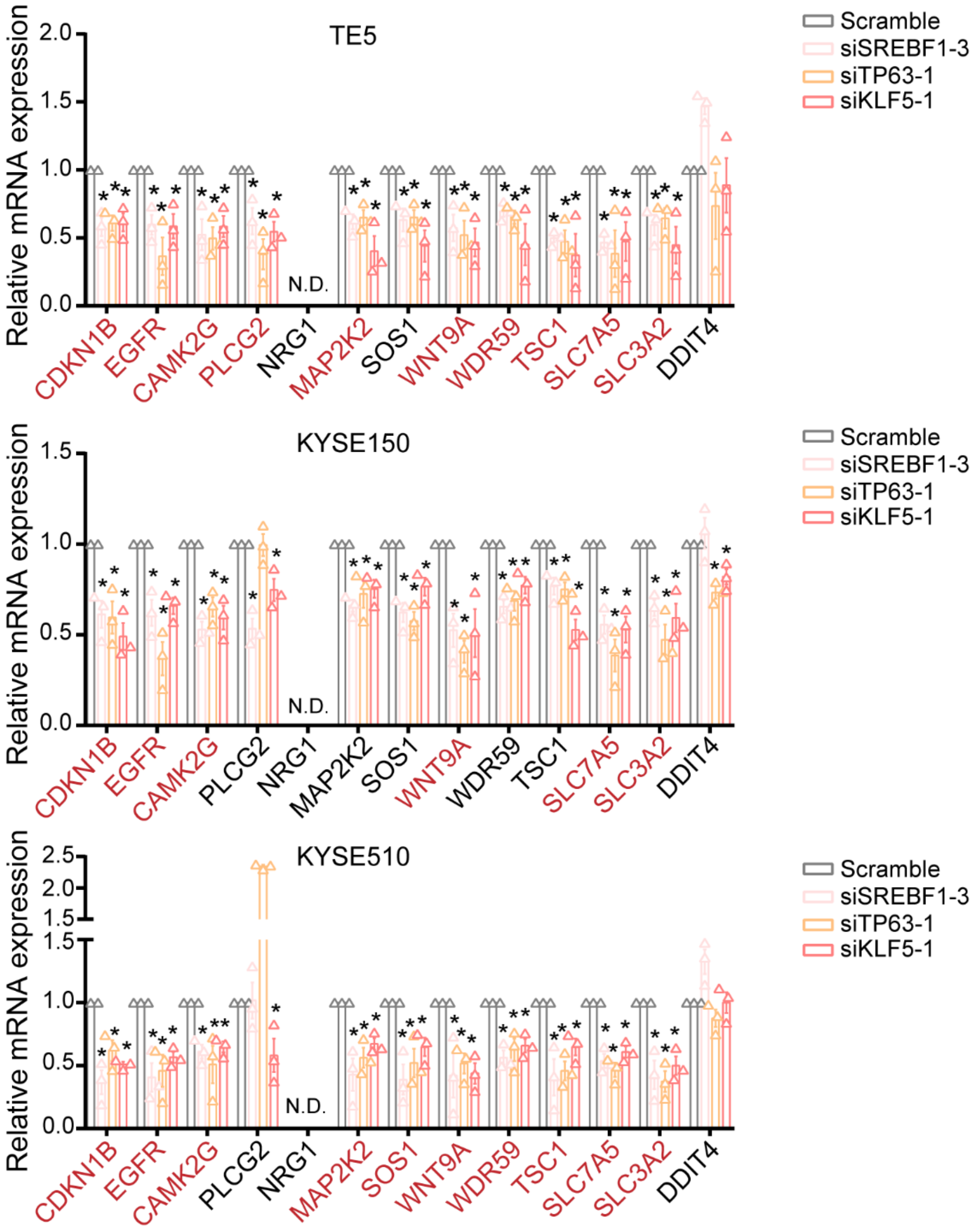

Supplementary Figure 9

TE5, KYSE150 and KYSE510 cells were transfected with 30 nM siRNA targeting either SREBF1, TP63 or KLF5 for 48 hours, and were then subjected to qRT-PCR experiments. Barplots displaying the fold changes relative to scramble control. Mean ±SEM are shown; n=3 (biological replicates); *, *P*<0.05, *P* values were determined by a two-sided t-test. N.D., not detectable.

Supplementary Tables

Supplementary Table 1. Relative mRNA expression of key genes for fatty acids/sphingolipids/Glycerophospholipids synthesis measured by RNA-seq

Gene name	Log_2 (Fold Change)	Gene name	Log_2 (Fold Change)
ACLY	-0.452	SMPD2	-0.014
FASN	-0.450	SMPD3	-0.853
SCD	-0.729	SMPD4	-0.048
FADS1	-0.266	DEGS1	0.024
FADS2	-0.432	DEGS2	-0.285
FADS3	-0.783	GBA	-0.021
SPTLC1	-0.324	GBA2	-0.028
SPTLC2	-0.401	SPHK2	0.240
SPTLC3	-0.367	GPAM	N/A
ELOVL1	0.370	GPAT2	-0.018
ELOVL2	1.074	GPAT3	0.135
ELOVL4	-0.669	GPAT4	0.096
ELOVL5	0.336	AGPAT2	-0.524
ELOVL6	-0.211	AGPAT5	-0.889
ELOVL7	-1.360	LPIN1	0.118
CERS1	-0.473	LPIN2	-0.219
CERS2	-0.131	LPIN3	-0.480
CERS5	0.031	CDS1	0.356
CERS6	-0.238	CDS2	-0.245
CERK	-1.911	CRLS1	N/A
SMPD1	-0.950	PTPMT1	-0.115

Supplementary Table 2. Univariate and multivariate analysis of factors associated with overall survival in 179 ESCC patients

Variables	Univariate analysis		Multivariate analysis	
	HR (95%CI)	*P* value	HR (95%CI)	*P* value
Age (>58 *vs* ≤58)	1.51 (1.05-2.18)	0.027	1.53 (1.04-2.24)	0.029
Gender (Female *vs* Male)	1.46 (0.87-2.45)	0.148	1.22 (0.722-2.06)	0.458
Histologic grade				
G2 *vs* G1	1.02 (0.60-1.74)	0.933	0.93 (0.54-1.59)	0.787
G3 *vs* G1	3.65 (1.55-8.59)	0.003	2.25 (0.92-5.51)	0.076
pTNM-stage (III+IV *vs* I+II)	2.04 (1.41-2.95)	0.000	2.19 (1.48-3.26)	0.000
SREBF1 (High *vs* Low) [a]	1.72 (1.06-2.79)	0.028	2.24 (1.36-3.71)	0.002

Note: Cox proportional hazards regression model. HR, hazard coefficient. [a], "Low" means IHC scores<127 ; "High" means IHC scores>126. *P* values were adjusted for multiple comparisons.

Supplementary Table 3. Primer sequences for quantitative RT-PCR

Gene name	Forward primer (5'-3')	Reverse primer (5'-3')
SREBF1	AGCTTCTCCATCAGTTCCAGC	TCAGAGAGGCCCACCACTTG
SREBF2	GGAGACCATGGAGACCCTCA	GACCTGGGTGAATGACCGTT
LXRA	ATGCAGCAAACAAGCTGGAAC	TTTTCCGCTTTTGTGGACGG
LXRB	CAGAGCGCAAGCGAAAGAAG	ATGAAAGCGTCCATCTGGCA
PPAGA	TCGGCGAGGATAGTTCTGGA	TGAAAGCGTGTCCGTGATGA
PGC1	TCTGAGAGGGCCAAGCAAAG	CTGTCTCCATCATCCCGCAG
PPARG	TACTGTCGGTTTCAGAAATGCC	GTCAGCGGACTCTGGATTCAG
HNF4A	GCAATGACACGTCCCCATCA	AGCCCGGAAGCATTTCTTGA
E2F	CTACGTGACGTGTCAGGACC	AAACATCGATCGGGCCTTGT
SOX5	TGCTCCAGCAACAGATCCAG	ATAGCTGAAGCCTGGAGGGA
TP63	CCTCGTCCACCAGTCCCTAT	AGGACACGTCGAAACTGTGC
KLF5	CCCTTGCACATACACCAATGC	GGATGGAGGTGGGGTTAAAT
SCD	TTCCTACCTGCAAGTTCTACACC	CCGAGCTTTGTAAGAGCGGT
ACLY	TGCTCTGAAATTGCCTTGG	CGGACTTCGGCAGAGGTAG
FASN	CAGGCACACACGATGGAC	CGGAGTGAATCTGGGTTGAT
ACSS2	AAAGGAGCAACTACCAACATCTG	GCTGAACTGACACACTTGGAC
CAMK2G	AGGCCTCACTTCCTTTGAGC	CTCCTAAAGCCCCATCCCGA
CDKN1B	TAATTGGGGCTCCGGCTAAC	AGAATCGTCGGTTGCAGGTC
EGFR	AGACAGCTTCTTGCAGCGAT	CAGGATTCTGCACAGAGCCA
NRG1	GAGTTGGCACCACAGCCTT	TGGCAGAGGCACTGTCATTT
PLCG2	GAAGCGATGAATGCGTCCAC	CCAGGATGAACACGGACGAA
WNT9A	CTATGCCATCTCCTCGGCTG	TGATCACCTTCACACCCACG
SOS1	CTGAAAAAGGTCCAGGGGCA	AAGGGTTTCTTCGCTTCCTCT
TSC1	CGAATATGTGGGCAAAGCCG	AGGCACCATGATGACAGACG
WDR59	ACCTTACAAGGCCACACTCG	TTCCATTTGACCTGGGAGGC
SLC7A5	CCCAACTTCTCATTTGAAGGCACC	CCATAGCGAAAGAGGCCGCTGTATAA
SLC3A2	AACATCCTGCTGTCCAACCC	CACTGGTAGACGATGGTGCC
DDIT4	CTTTGGGACCGCTTCTCGT	GGTAAGCCGTGTCTTCCTCC
MAP2K2	TCATCATGGCCAGGAAGCTG	CCTCTTGGCCTCTTTCAGCA
Actin	AGCGAGCATCCCCCAAAGTT	GGGCACGAAGGCTCATCATT

Supplementary Table 4. Primer sequences for constructing pGL3-promoter luciferase vectors

Name	Forward primer (5'-3')	Reverse primer (5'-3')
Negative control	CGCCAATGGACACTCACA	CCCTCTGGTGCTTCTTCC
SREBF1-promoter	GCACTGCTGCCCCTGGTT	TGTCCCGTGTTAGCCCTTCC
SREBF1-E1	CGTTGGCCCTACCCCTCC	GCAGACGCTGGCACCGAG
SREBF1-E2	ACTGTATCCCTTGTAGCCC	AATGGTGCCTGCCTCCTG
SREBF1-E3	CAGCAGGCAGATAGTCAC	CAGTAATGGGTAGAAACAAA
TP63-T1	ATCACAAGCCATACTTACTC	CTCAAACTCCAACAGGGT
TP63-T2	GGTATGAGGGAGACAAGA	TCAAAGTGAATTAGGGAC
TP63-T3	AGCTTCCTCTTTGTACCCATAA	GCCATCCTTCGGCTTTCT
KLF5-K1	CCCCTACCTAGCTGCCTTCT	AAACTCTTCCGCTCTTCCACA

Supplementary Table 5. siRNA, shRNA and sgRNA sequences

Name	Sense sequence (5' - 3')	Antisene sequence (5' - 3')
siSREBF1-1	CCCACUCCAUUGAAGAUGUTT	ACAUCUUCAAUGGAGUGGGTT
siSREBF1-3	GCCUGACCAUCUGUGAGAATT	UUCUCACAGAUGGUCAGGCTT
shSREBF1-3	GCCTGACCATCTGTGAGA	UCUCACAGAUGGUCAGGU
shTP63-1	CCGTTTCGTCAGAACACACAT	AUGUGUGUUCUGACGAAACGG
shTP63-2	GGACAGCAGCATTGATCAA	UUGAUCAAUGCUGCUGUCC
siTP63-1	CCGUUUCGUCAGAACACACAUTT	AUGUGUGUUCUGACGAAACGGTT
siTP63-2	GGACAGCAGCAUUGAUCAATT	UUGAUCAAUGCUGCUGUCCTT
siKLF5-1	CGAUUACCCUGGUUGCACATT	UGUGCAACCAGGGUAAUCGCATT
siKLF5-2	GAUGUGAAAUGGAGAAGUATT	UACUUCUCCAUUUCACAUCTT
Negative control-sgRNA	CACCGAGCTGTTCGTTACCT	
Promoter-sgRNA-1	GGCACCGGAAGGGCTAACAC	
Promoter-sgRNA-2	TAACACGGGACAGCCCCACC	
E1-sgRNA-1	CAACGGCCTGGACGCCCCAA	
E1-sgRNA-2	CGACTTCACCTGTCAAGGCG	
E2-sgRNA-1	GGATAAGTCTCCTCTTGAGG	
E2-sgRNA-2	GGCTACAAGGGATACAGTCT	
E3-sgRNA-1	TATAAGTTTCGACCTAGACC	
E3-sgRNA-2	TTATAAGTTTCGACCTAGAC	
