# Supplementary material for: Interplay and cooperation between SREBF1 and master transcription factors regulate lipid metabolism and tumor-promoting pathways in squamous cancer
Source: Nat Commun. 2021 Jul 16;12:4362. doi: 10.1038/s41467-021-24656-x (PMC8285542; doi:10.1038/s41467-021-24656-x)
Supplement: Supplementary file 3 — Description of Additional Supplementary Files [file 41467_2021_24656_MOESM3_ESM.pdf]

Description of Additional Supplementary Files

Supplementary Data 1. The genes positively or negatively correlated with TP63 in SCC

Supplementary Data 2. 4C-Seq using SREBF1 promoter as the bait in TE5 cells

Supplementary Data 3. Source data of lipidomics

Supplementary Data 4. The top 15 most significantly enriched motifs by sequence motif enrichment analysis performed in separately promoter and distal regions for each group peaks

Supplementary Data 5. The exact P values of all statistical tests
