# Supplementary material for: Interplay and cooperation between SREBF1 and master transcription factors regulate lipid metabolism and tumor-promoting pathways in squamous cancer
Source: Nat Commun. 2021 Jul 16;12:4362. doi: 10.1038/s41467-021-24656-x (PMC8285542; doi:10.1038/s41467-021-24656-x)
Supplement: Supplementary file 9 — Reporting summary [file 41467_2021_24656_MOESM9_ESM.pdf]

nature research

Corresponding author(s): De-Chen Lin

Last updated by author(s): 2021-06-02

Reporting Summary

Nature Research wishes to improve the reproducibility of the work that we publish. This form provides structure for consistency and transparency in reporting. For further information on Nature Research policies, see our Editorial Policies and the Editorial Policy Checklist.

Statistics

For all statistical analyses, confirm that the following items are present in the figure legend, table legend, main text, or Methods section.

n/a	Confirmed	
☐	☒	The exact sample size (*n*) for each experimental group/condition, given as a discrete number and unit of measurement
☐	☒	A statement on whether measurements were taken from distinct samples or whether the same sample was measured repeatedly
☐	☒	The statistical test(s) used AND whether they are one- or two-sided<br>*Only common tests should be described solely by name; describe more complex techniques in the Methods section.*
☐	☒	A description of all covariates tested
☐	☒	A description of any assumptions or corrections, such as tests of normality and adjustment for multiple comparisons
☐	☒	A full description of the statistical parameters including central tendency (e.g. means) or other basic estimates (e.g. regression coefficient) AND variation (e.g. standard deviation) or associated estimates of uncertainty (e.g. confidence intervals)
☐	☒	For null hypothesis testing, the test statistic (e.g. *F*, *t*, *r*) with confidence intervals, effect sizes, degrees of freedom and *P* value noted<br>*Give P values as exact values whenever suitable.*
☒	☐	For Bayesian analysis, information on the choice of priors and Markov chain Monte Carlo settings
☒	☐	For hierarchical and complex designs, identification of the appropriate level for tests and full reporting of outcomes
☐	☒	Estimates of effect sizes (e.g. Cohen's *d*, Pearson's *r*), indicating how they were calculated

Our web collection on statistics for biologists contains articles on many of the points above.

Software and code

Policy information about availability of computer code

Data collection

The Cancer Genome Atlas Network (TCGA, GDC v16.0) data was downloaded using TCGAbiolinks (V2.14.1) R package. The blacklisted regions were downloaded from ENCODE database (https://sites.google.com/site/anshulkundaje/projects/blacklists). Molecular Signatures Database v7.4 (https://www.gsea-msigdb.org/gsea/msigdb/index.jsp) were used to do the GSEA (v3.0) (https://www.gsea-msigdb.org/gsea/index.jsp). R version is 3.6.3..

Data analysis

ChIP-Seq data were analyzed by Bowtie2 (v2.2.6, k=2) based on human reference genome (HG19), and Picard MarkDuplicates (v 1.136), MACS2 (v2.1.2) and DeepTools (v3.1.3).
The bigwig files were visualized in Integrative Genomics Viewer (IGV, v2.5.0).
ROSE (Rank Order of Super Enhancers) was used to identify typical-enhancers, and super-enhancers were then classified using a cutoff at the inflection point (tangent slope=1) based on the ranking order.
HOMER findMotifsGenome.pl function was used to identify enriched motifs in selected regions. The parameters in HOMER are hg19 -size 200 -len 8,10,12.
The RNA-Seq data of siSREBF1, siTP63 and siKLF5 were generated in TE5 and KYSE150 cell lines.
Trim Galore (v 0.4.1) was used to remove the adapters.
49 bp single-end and 150 bp paried-end reads were aligned to human reference genome (HG19) using STAR (v3.5.1b) (--alignIntronMin 20 --alignIntronMax 1000000 --alignSJoverhangMin 8 --quantMode GeneCounts) method.
DESeq2 (v1.26.0) was used to identify the differentially expressed genes based on the read counts.
For GSEA analysis, we first ranked tumor samples based on the expression of TP63, and classified the samples into two groups (top and bottom 30% samples). Secondly, differentially expressed genes were determined using limma (v3.42.2) R package. GSEA Preranked (v3.0) method was performed to identify the Hallmark pathways and motifs in Molecular Signatures Database v7.4 (https://www.gsea-msigdb.org/gsea/msigdb/index.jsp).
The clusterProfiler (v3.14.3) R package was used to perform KEGG pathway analysis.
The analyses for IHC were performed with SPSS for Windows ver.18.0 software (SPSS Institute, Chicago, IL, USA). Kaplan-Meier curve was

constructed for overall survival analysis using a Log-rank test. Each P value is two-tailed and significance level is 0.05. For comparisons of continuous variables between groups, two-tailed Student t test was used. The values at P<0.05 (*) and P<0.01 (**) were considered statistically significant. Diagrams were created by GraphPad Prism software and data were shown as the mean ± SEM.

For manuscripts utilizing custom algorithms or software that are central to the research but not yet described in published literature, software must be made available to editors and reviewers. We strongly encourage code deposition in a community repository (e.g. GitHub). See the Nature Research guidelines for submitting code & software for further information.

Data

Policy information about availability of data

All manuscripts must include a data availability statement. This statement should provide the following information, where applicable:

- Accession codes, unique identifiers, or web links for publicly available datasets
- A list of figures that have associated raw data
- A description of any restrictions on data availability

Data availability.
The mRNA expression (RNA-Seq level 3 data) data of 23 types of cancers were retrieved from the datasets produced by The Cancer Genome Atlas Network (TCGA, GDC v16.0) using TCGAbiolinks (V2.14.1) R package.
Microarray RNA expression data of ESCC and LUSC, were retrieved from GEO database (GSE53624, SRP064894 and GSE4573).
ChIP-Seq data of SREBF1 in MCF7 and HepG2 cell lines were from ENCODE database (https://www.encodeproject.org/).
H3K27Ac ChIP-Seq data in 8 ESCC cell lines (TE5, TE7, KYSE70, KYSE150, KYSE140, KYSE180, KYSE200 and KYSE510) were collected from our previous studies (Jiang, Y. et al. Nature communications 9, 3619 (2018)).
H3K4Me3, TP63 and SOX2 ChIP-Seq in TE5 cell line were collected from our previous studies (Jiang, Y. et al. Nature communications 9, 3619 (2018) and Jiang, Y.Y. et al. Gastroenterology 159, 1311-1327 (2020)).
The blacklisted regions were downloaded from ENCODE database (https://sites.google.com/site/anshulkundaje/projects/blacklists).
Molecular Signatures Database v7.4 (https://www.gsea-msigdb.org/gsea/msigdb/index.jsp) were used to do the GSEA (v3.0) (https://www.gsea-msigdb.org/gsea/index.jsp).
The ChIP-Seq and RNA-Seq datasets generated in this study have been deposited in the Gene Expression Omnibus (GEO) repository with the accession code GSE143803.
The remaining data are available in the article or Supplementary Information files, or available from the authors upon request.

Field-specific reporting

Please select the one below that is the best fit for your research. If you are not sure, read the appropriate sections before making your selection.

[X] Life sciences   [ ] Behavioural & social sciences   [ ] Ecological, evolutionary & environmental sciences

For a reference copy of the document with all sections, see nature.com/documents/nr-reporting-summary-flat.pdf

Life sciences study design

All studies must disclose on these points even when the disclosure is negative.

Sample size	We didn't perform computation to pre-determine the sample size for the perturbation experiments. We justified the sample size based on published papers, our experience and those generally employed in the field. The results obtained suggest the chosen sample size is appropriate because either clear distinctions are observed or the results have reached statistical significance.
Data exclusions	No data was excluded.
Replication	The number of the repeats for experiments was described in the corresponding figure legends.
Randomization	At the initiation of in vivo experiment, mice were randomly assigned to either experimental or control groups. For the experiments in vitro, no method of randomization was used as we utilized all available data to maximize the sample size and none of the experiments involved allocation of samples to test groups.
Blinding	No blinding was required in our study as no group allocation was involved in this study.

Reporting for specific materials, systems and methods

We require information from authors about some types of materials, experimental systems and methods used in many studies. Here, indicate whether each material, system or method listed is relevant to your study. If you are not sure if a list item applies to your research, read the appropriate section before selecting a response.

Materials & experimental systems

n/a	Involved in the study
☐	☒ Antibodies
☐	☒ Eukaryotic cell lines
☒	☐ Palaeontology and archaeology
☐	☒ Animals and other organisms
☐	☒ Human research participants
☒	☐ Clinical data
☒	☐ Dual use research of concern

Methods

n/a	Involved in the study
☐	☒ ChIP-seq
☒	☐ Flow cytometry
☒	☐ MRI-based neuroimaging

Antibodies

Antibodies used

Anti-SREBF1 (Proteintech, 14088-1-AP, 1:1000 for western blotting and 4 µg for ChIP), anti-KLF5 (Santa Cruz Biotechnology, sc-398409X, 1:1000 for western blotting and 4 µg for ChIP), anti-TP63 (R&D Systems, AF1916-SP, 1:1000), anti-Actin (Santa Cruz Biotechnology, sc-8432, 1:2000), anti-ACLY (Cell Signaling Technology, 4332, 1:1000), Anti-FASN (Cell Signaling Technology, 3180, 1:1000), anti-GAPDH (Cell Signaling Technology, 2118, 1:2000), anti-mTOR (Cell Signaling Technology, 2972S, 1:1000), anti-Phospho-mTOR (S2448) (Cell Signaling Technology, 5536T, 1:1000), anti-Phospho-MEK1 (Ser298) (Cell Signaling Technology, 9128S, 1:1000), anti-MEK1/2(D1A5) Rabbit (Cell Signaling Technology, 9124S, 1:1000) , anti-p70 S6 Kinase (Cell Signaling Technology, 9202S, 1:1000), anti-Phospho-p70 S6 Kinase (Cell Signaling Technology, 9205S, 1:1000), anti-mouse IgG-HRP (Jackson ImmunoResearch Laboratories, Inc., 115-035-003, 1:10000), anti-rabbit IgG-HRP (Jackson ImmunoResearch Laboratories, Inc., 111-035-144, 1:10000), anti-goat IgG-HRP (Jackson ImmunoResearch Laboratories, Inc., 705-035-003, 1:10000).

Validation

SREBF1 antibodies are validated for western blotting in numerous publications (PMID: 29858247, 29084766).
KLF5 antibodies are validated for western blotting and ChIP in publication (PMID: 31409603).
TP63 antibodies are validated for IHC in publications (PMID: 31968252; PMID: 33050277).
Actin antibodies are validated for western blotting in publications (PMID: 33539814; PMID: 33561012).
ACLY antibodies are validated for western blotting in publications (PMID: 32849657; PMID: 32579936).
FASN antibodies are validated for western blotting in publications (PMID: 33166087; PMID: 33171690).
GAPDH antibodies are validated for western blotting in publications (PMID: 33479769; PMID: 33465556).
mTOR antibodies are validated for western blotting in publications (PMID: 33635313; PMID: 33425737).
Phospho-mTOR antibodies are validated for western blotting in publications (PMID: 33179755; PMID: 33168829).
MEK1/2 antibodies are validated for western blotting in publications (PMID: 29862663; PMID: 22102825).
Phospho-MEK1/2 antibodies are validated for western blotting in publications (PMID: 27613601; PMID: 26257058).
p70 S6 antibodies are validated for western blotting in publications (PMID: 33635313; PMID: 33514739).
Phospho-p70 S6 antibodies are validated for western blotting in publications (PMID: 33275593; PMID: 33257668).
Mouse IgG-HRP antibodies are validated for western blotting in publications (PMID: 33720931; PMID: 33720931).
Rabbit IgG-HRP antibodies are validated for western blotting in publications (PMID: 33672589; PMID: 33516944).
Goat IgG-HRP antibodies are validated for western blotting in publications (PMID: 33060128; PMID: 33298866).

Eukaryotic cell lines

Policy information about cell lines

Cell line source(s)

TE5 cell line was provided by Dr Koji Kono from Cancer Science Institute of Singapore.
KYSE150, KYSE180 and KYSE510 cell lines were provided by Dr Y Shimada from Kyoto University.
UMSCC1 cell line was provided by Dr Timothy Chan from Memorial Sloan Kettering Cancer Center.

Authentication

All cell lines were recently authenticated by Short Tandem Repeat (STR) analysis.

Mycoplasma contamination

All cell lines used in-house tested negative for mycoplasma.

Commonly misidentified lines
(See ICLAC register)

No cell lines from the ICLAC register were used.

Animals and other organisms

Policy information about studies involving animals; ARRIVE guidelines recommended for reporting animal research

Laboratory animals

Five-week-old male NU/NU mice were purchased from Vital River Laboratories (Beijing, China). Mice were housed at 1 or 5 mice per cage. The macroenvironment of the animal housing room was maintained at 22.2 ± 1 °C (72 °F) and 30% to 40% humidity with at least 12 fresh-air changes hourly and a controlled 14:10-hours light:dark cycle.

Wild animals

No wild animals were used in the study.

Field-collected samples

No field-collected samples were used in the study.

Ethics oversight

All animal studies were conducted in accordance with protocols approved by the Animal Research Committee of the Shantou

Administration Center of Shantou University.

Note that full information on the approval of the study protocol must also be provided in the manuscript.

Human research participants

Policy information about studies involving human research participants

Population characteristics	The total of 179 ESCC patients included 30 females and 149 males at the mean age of 59 (the age range from 39 to 88). All specimens were confirmed as ESCC by pathologists in the Clinical Pathology Department of the hospital. Only primary samples from surgical patients with written informed consent were included.
Recruitment	The formalin-fixed, paraffin embedded tissue specimens for Immunohistochemistry (IHC) were collected from primary ESCC patients undergoing curative resection at the Shantou Central Hospital, including 179 patients treated during November 2007 to January 2011. There was no selection bias.
Ethics oversight	Ethical approval was obtained from the ethical committee of the Central Hospital of Shantou City and the ethical committee of the Medical College of Shantou University.

Note that full information on the approval of the study protocol must also be provided in the manuscript.

ChIP-seq

Data deposition

☒ Confirm that both raw and final processed data have been deposited in a public database such as GEO.

☒ Confirm that you have deposited or provided access to graph files (e.g. BED files) for the called peaks.

Data access links *May remain private before publication.*	https://www.ncbi.nlm.nih.gov/geo/query/acc.cgi?acc=GSE143803
Files in database submission	Accession "GSE143803" have been released at the GEO database.
Genome browser session (e.g. UCSC)	Not applicable.

Methodology

Replicates	ChIP-Seq data of SREBF1 were generated in both TE5 and KYSE150 cell lines. ChIP-Seq data of KLF5 was generated in TE5 cell line. No replicate was performed.
Sequencing depth	For the SREBF1 ChIP-Seq data in KYSE150 cell line, the sequencing depth data is ~21.44M; the total reads and the uniquely mapped genes are 21,444,569 and 12,345,507, respectively; the length of reads is 150bp (paired-end).<br>For the SREBF1 ChIP-Seq data in TE5 cell line, the sequencing depth data is ~21.35M; the total reads and the uniquely mapped genes are 21,350,700 and 16,803,360, respectively; the length of reads is 150bp (paired-end).<br>For the KLF5 ChIP-Seq data in TE5 cell line, the sequencing depth data is ~22.11M; the total reads and the uniquely mapped genes are 22,107,646 and 18,856,511, respectively; the length of reads is 150bp (paired-end).
Antibodies	Anti-SREBF1 (Proteintech, 14088-1-AP, 4 µg for each ChIP experiment), anti-KLF5 (Santa Cruz Biotechnology, sc-398409X, 4 µg for each ChIP experiment).
Peak calling parameters	150 bp paired-end reads were aligned to human reference genome (HG19) using Bowtie2 (v2.2.6) (k=2). Then we used Picard MarkDuplicates tool to mark PCR duplicates. ENCODE blacklisted regions were removed (https://sites.google.com/site/anshulkundaje/projects/blacklists). Macs2 was utilized to identify the peaks with the parameters --bdg --SPMR --nomodel --extsize 200 -q 0.01.
Data quality	Macs2 was utilized to identify the peaks with the parameters --bdg --SPMR --nomodel --extsize 200 -q 0.01. The number of peaks in KYSE150 SREBF1 ChIP-Seq data is 1,654 with the q < 0.01. The number of peaks in TE5 SREBF1 ChIP-Seq data is 2,291 with the q < 0.01. The number of peaks in TE5 KLF5 ChIP-Seq data is 5,466 with the q < 0.01.
Software	ChIP-Seq data were analyzed by Bowtie2 (v2.2.6, k=2) based on human reference genome (HG19), and Picard MarkDuplicates (v 1.136), MACS2 (v2.1.2) and DeepTools (v3.1.3).
