# Supplementary material for: Interplay and cooperation between SREBF1 and master transcription factors regulate lipid metabolism and tumor-promoting pathways in squamous cancer
Source: Nat Commun. 2021 Jul 16;12:4362. doi: 10.1038/s41467-021-24656-x (PMC8285542; doi:10.1038/s41467-021-24656-x)

Figure 2e right panel

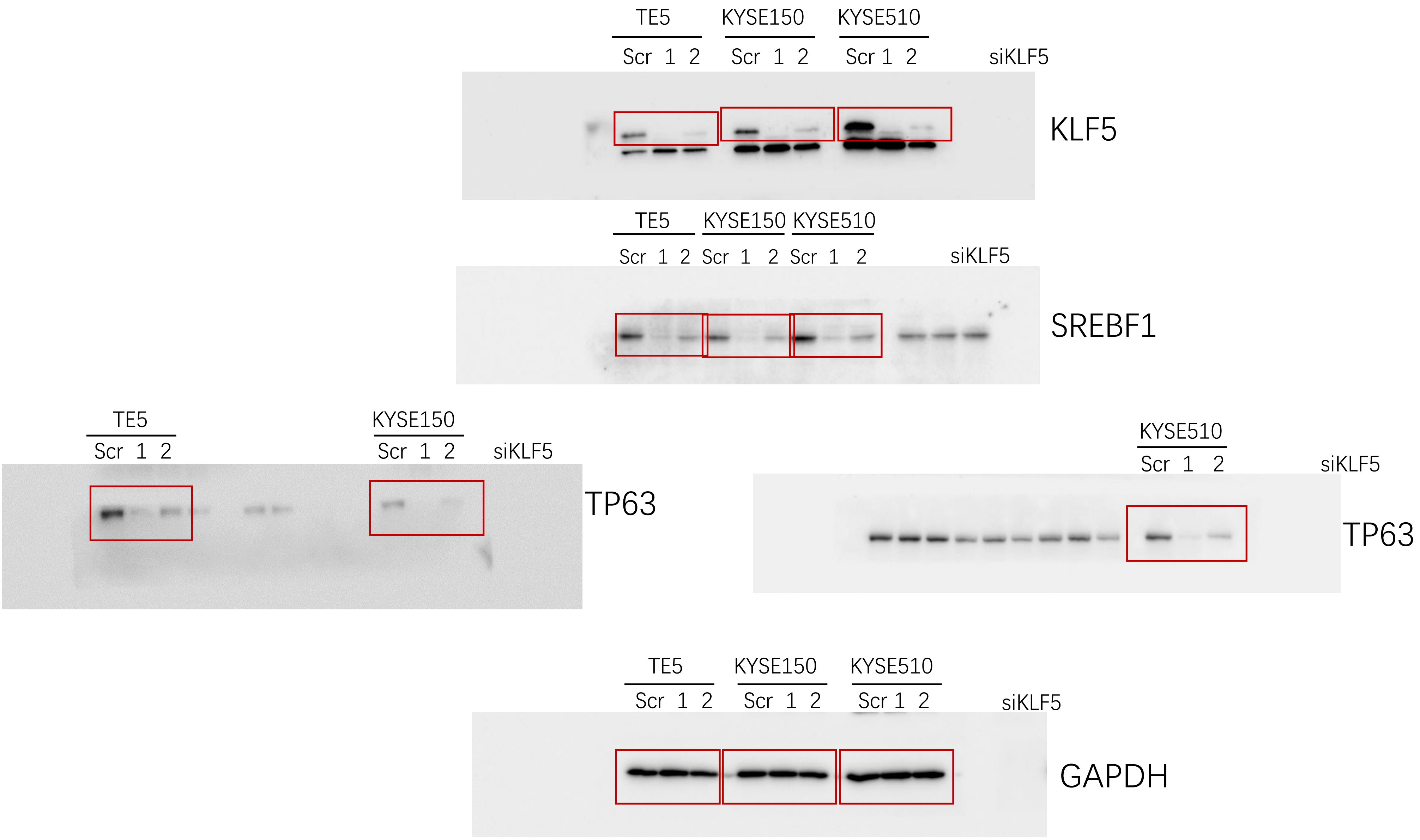

Figure 2f right panel

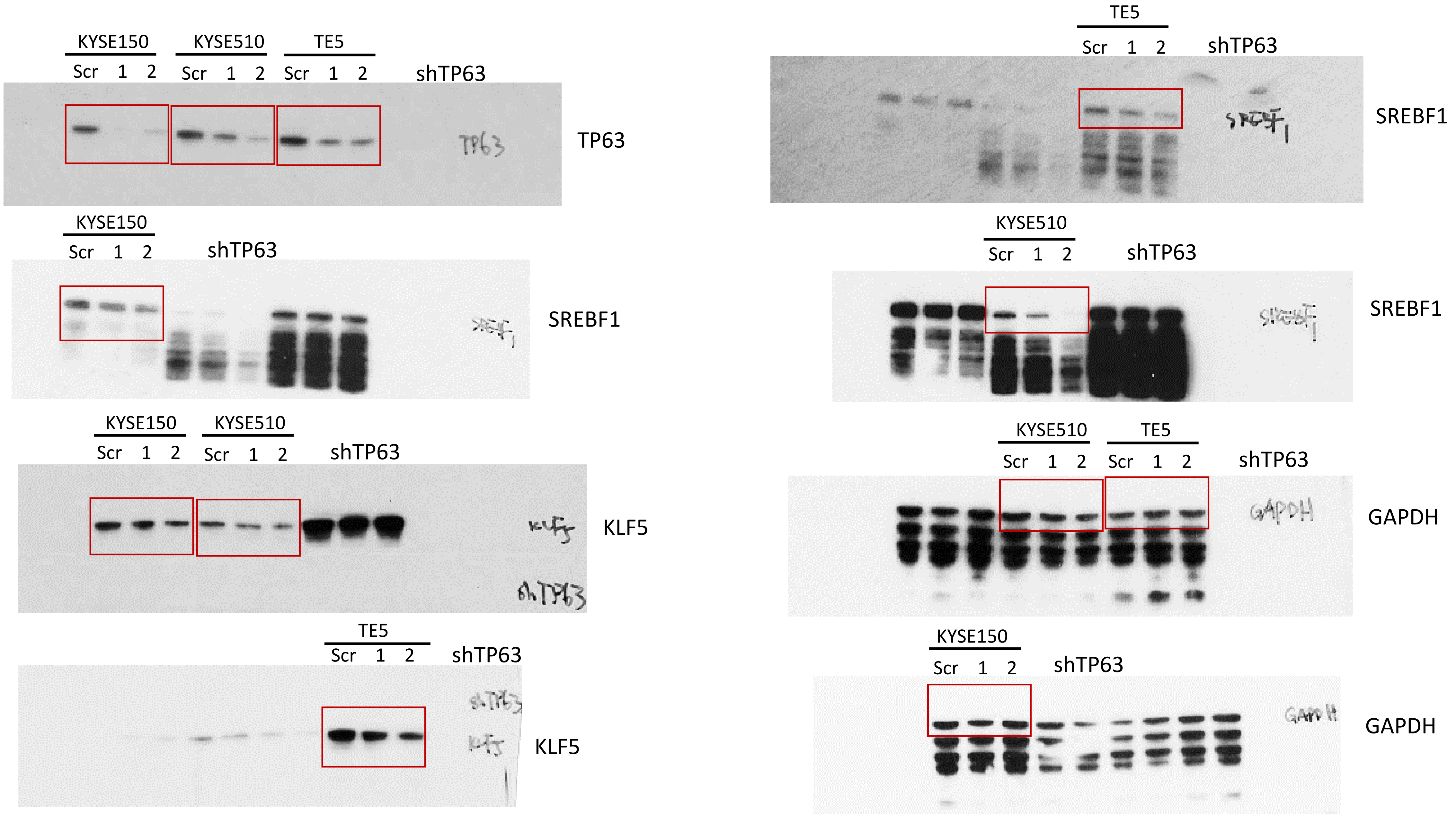

Figure 3e bottom panel

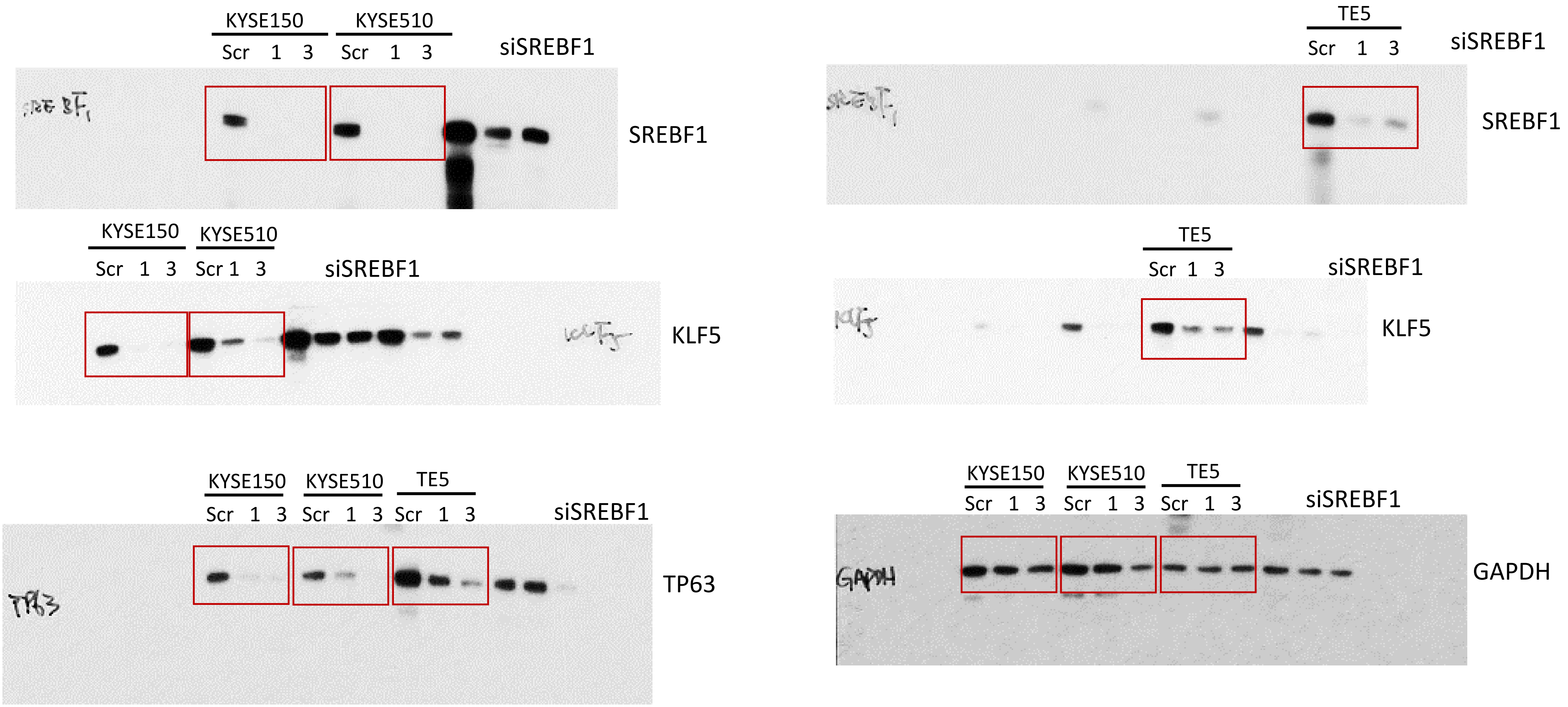

Figure 4b

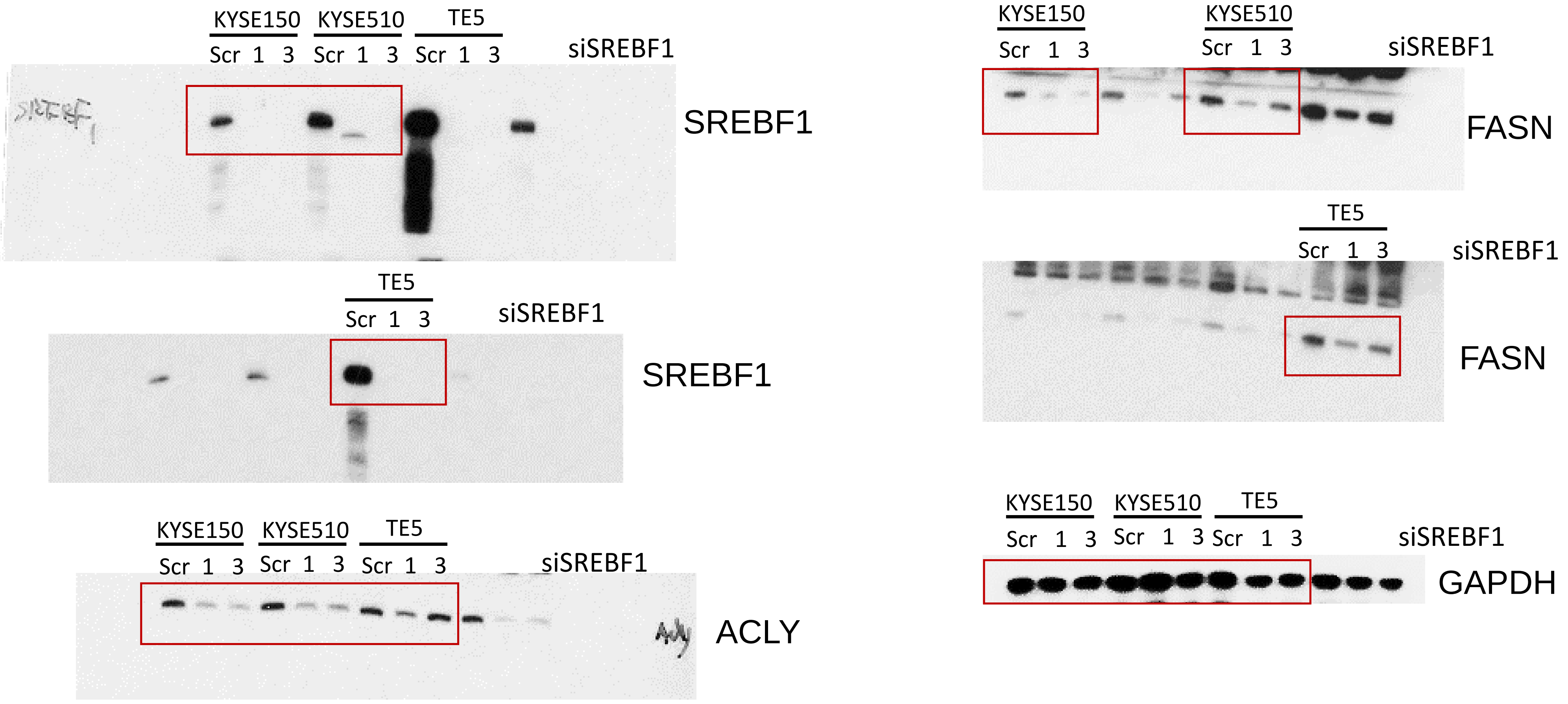
KYSE150 KYSE510 TE5
Scr 1 3 Scr 1 3 Scr 1 3
siSREBF1
SREBF1
TE5
Scr 1 3
siSREBF1
SREBF1
KYSE150 KYSE510 TE5
Scr 1 3 Scr 1 3 Scr 1 3
siSREBF1
ACLY
KYSE150 KYSE510
Scr 1 3 Scr 1 3
siSREBF1
FASN
TE5
Scr 1 3
siSREBF1
FASN
KYSE150 KYSE510 TE5
Scr 1 3 Scr 1 3 Scr 1 3
siSREBF1
GAPDH

Figure 4c-KYSE510

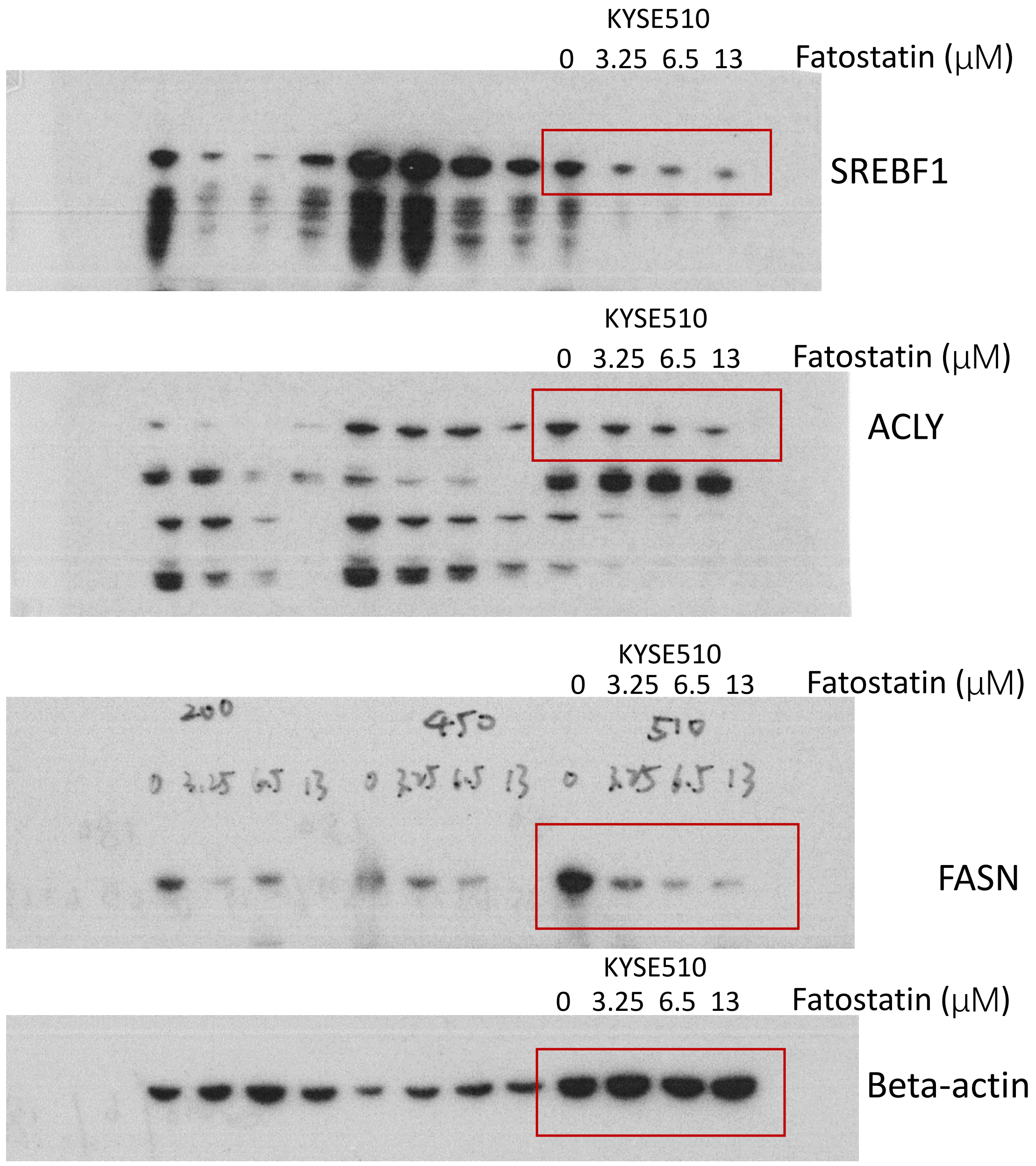

Figure 4c-KYSE150

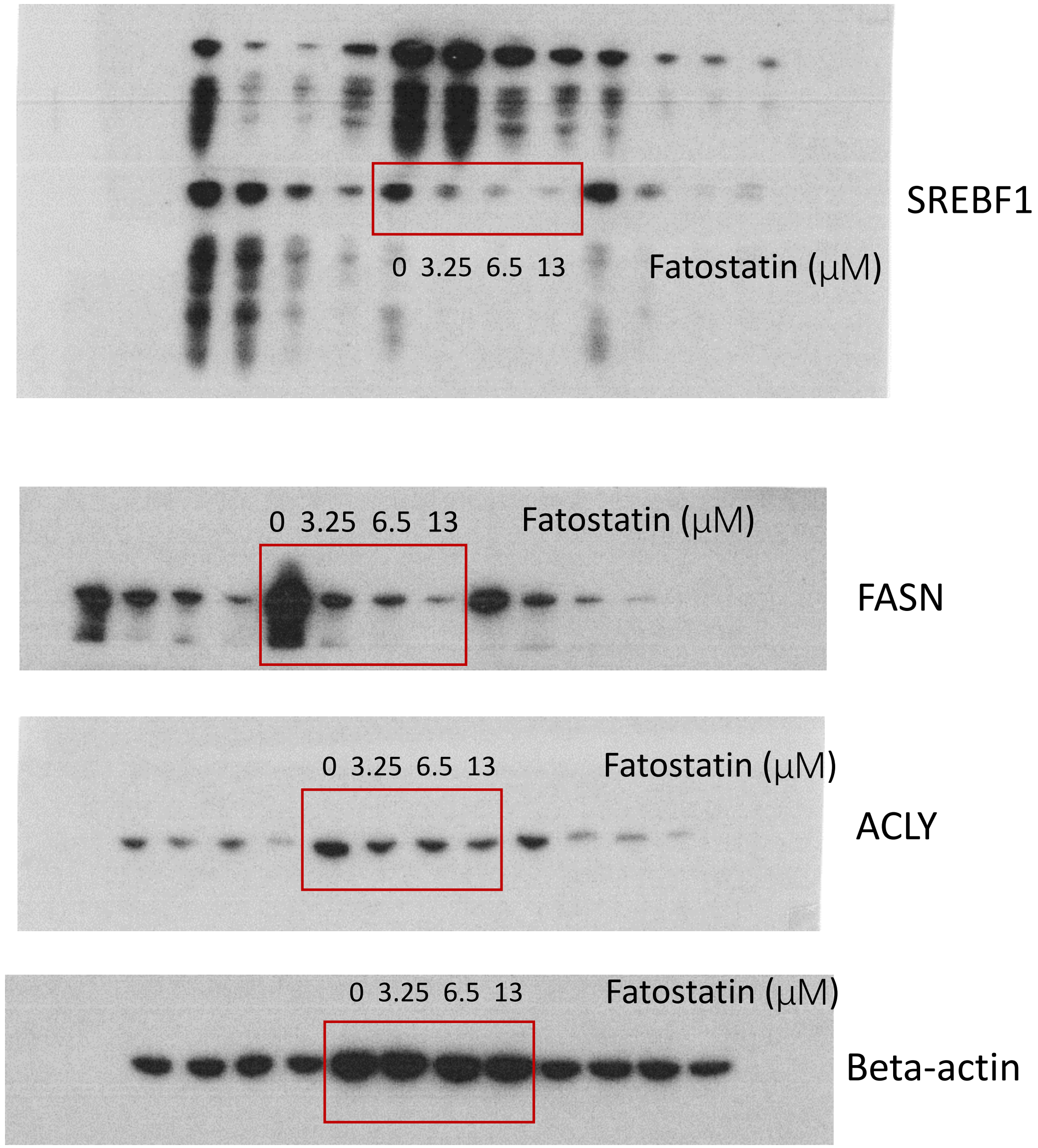

Figure 4c-TE5

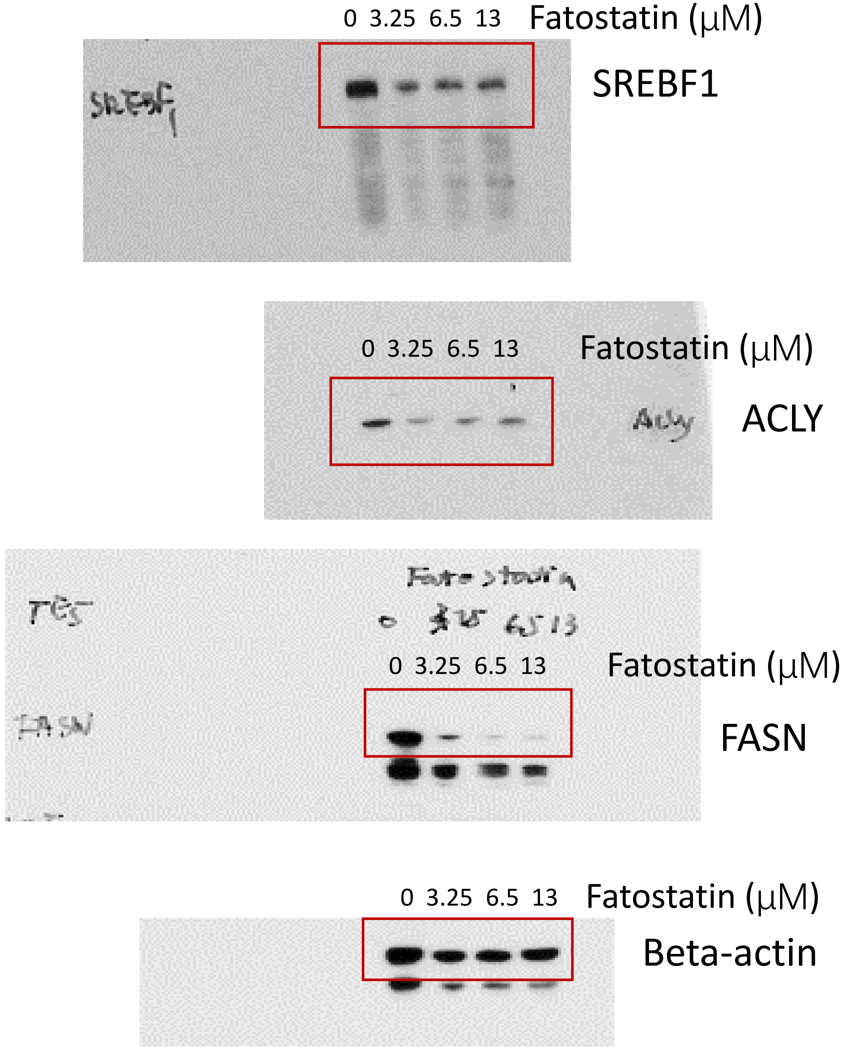

Figure 7e

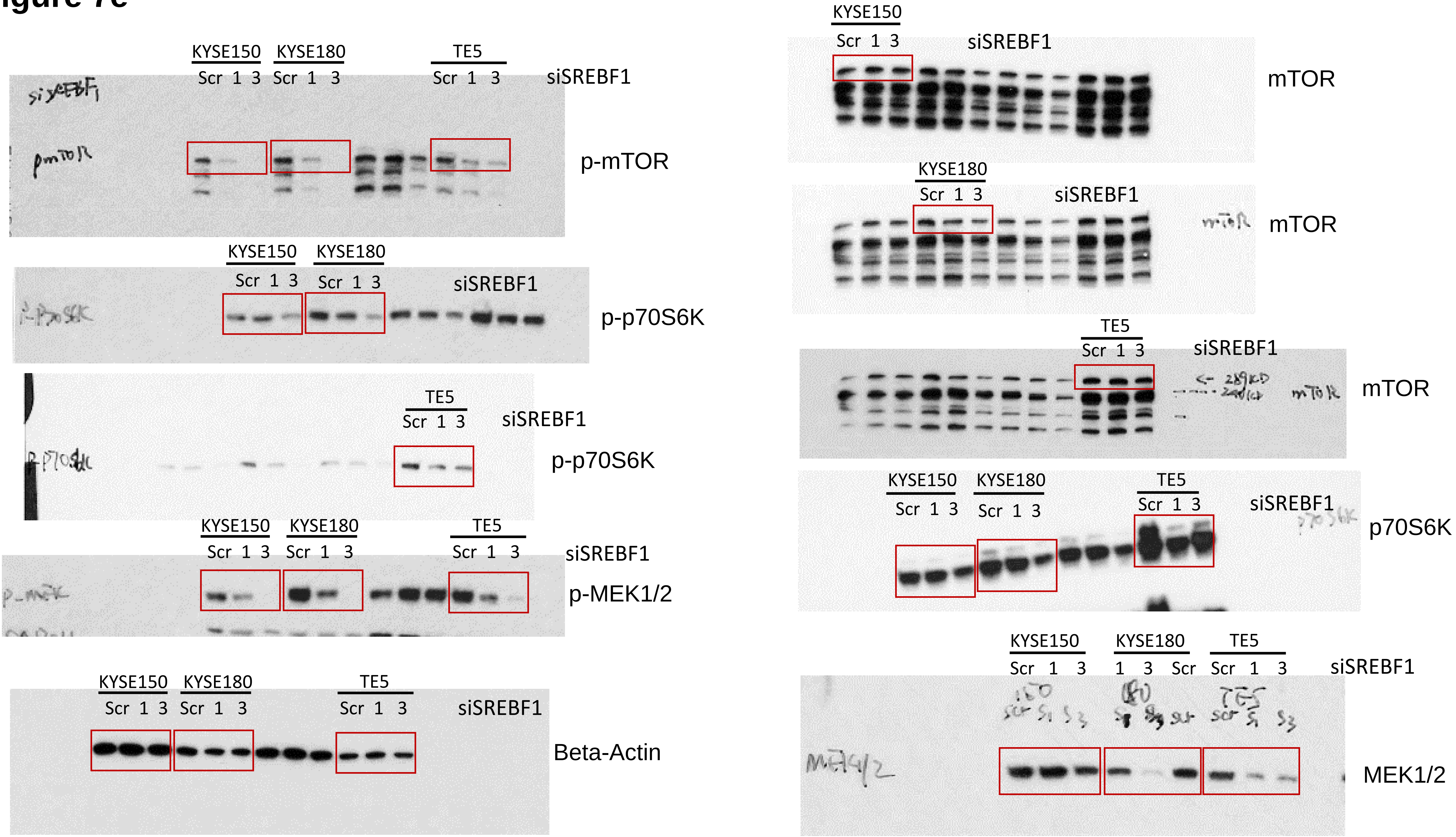
KYSE150
KYSE180
TE5
Scr 1 3
siSREBF1
p-mTOR
p-p70S6K
p-MEK1/2
Beta-Actin
mTOR
p70S6K
MEK1/2

Supplementary Figure 4b

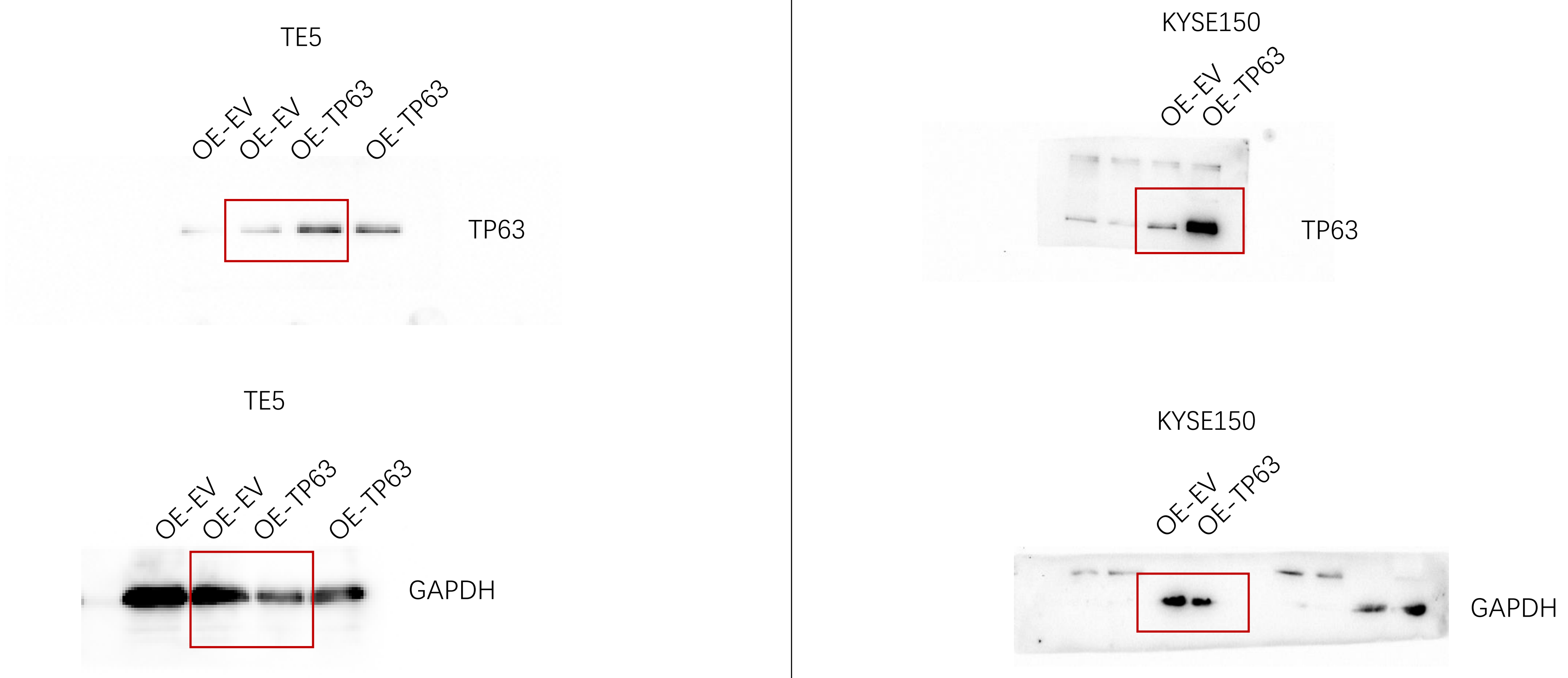

Supplementary Figure 5b

TE5

OE-EV OE-EV OE-SREBF1 OE-SREBF1

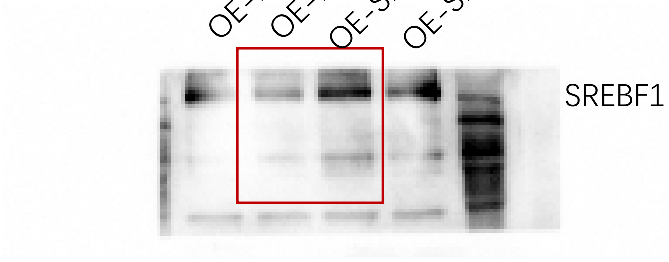

SREBF1

TE5

OE-EV OE-EV OE-SREBF1 OE-SREBF1

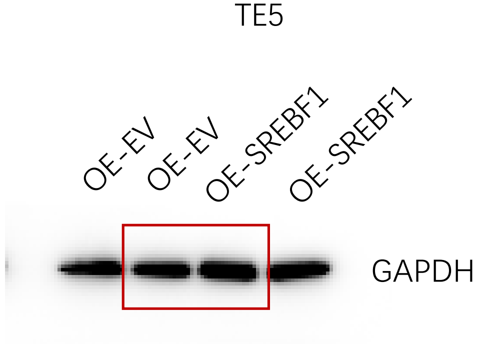

GAPDH

KYSE150

OE-EV OE-SREBF1

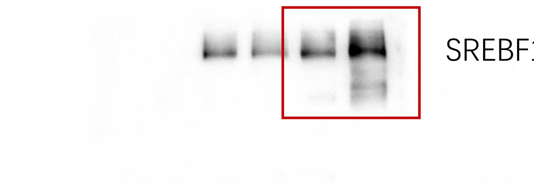

SREBF1

KYSE150

OE-EV OE-SREBF1

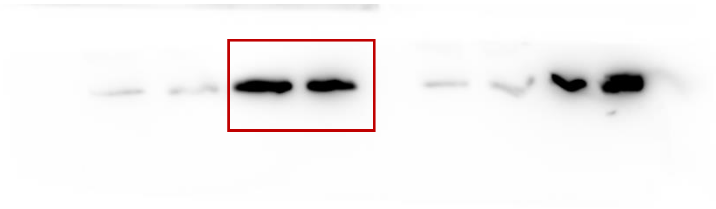

GAPDH

Supplementary Figure 6b

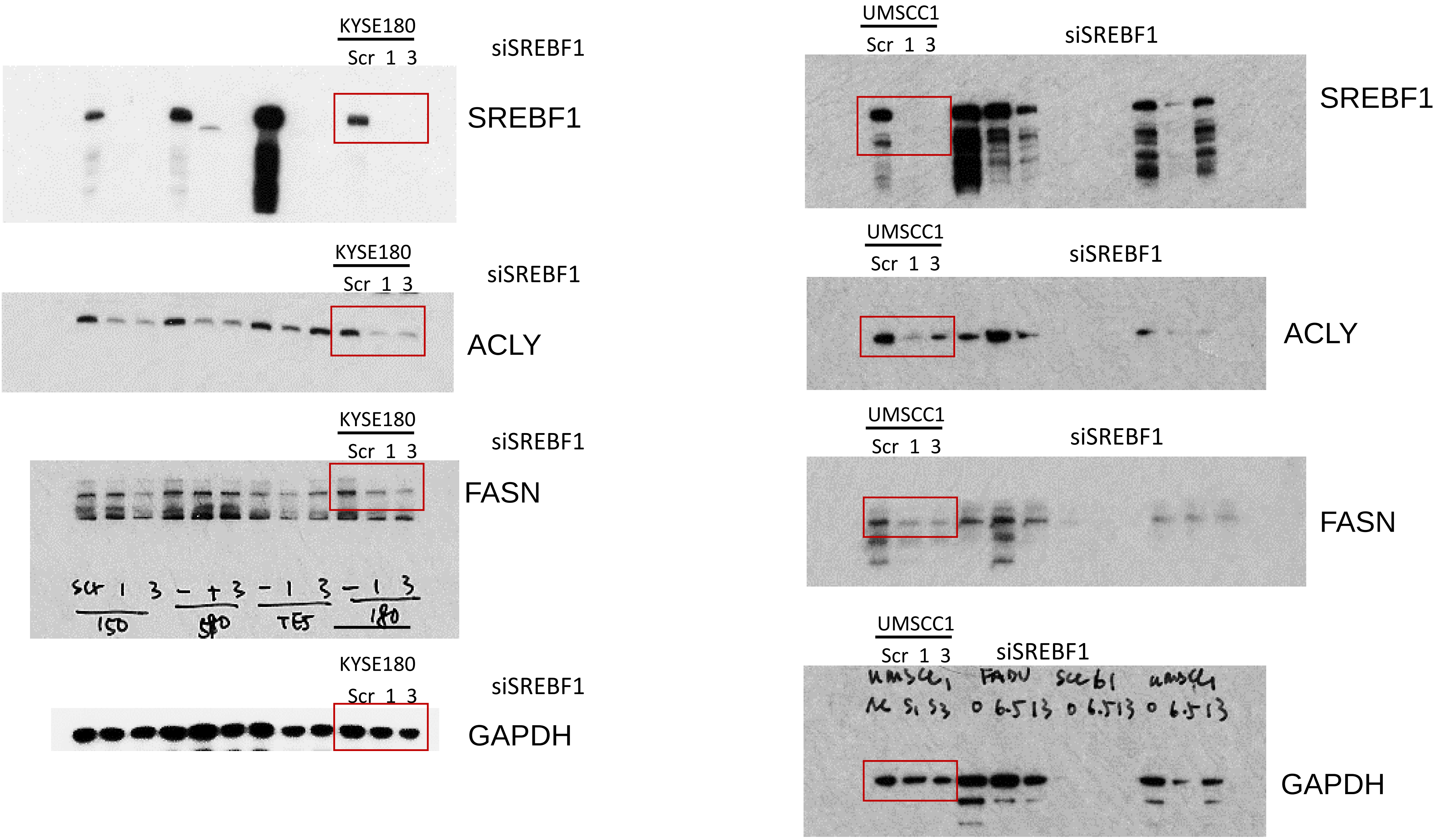

Supplementary Figure 6c

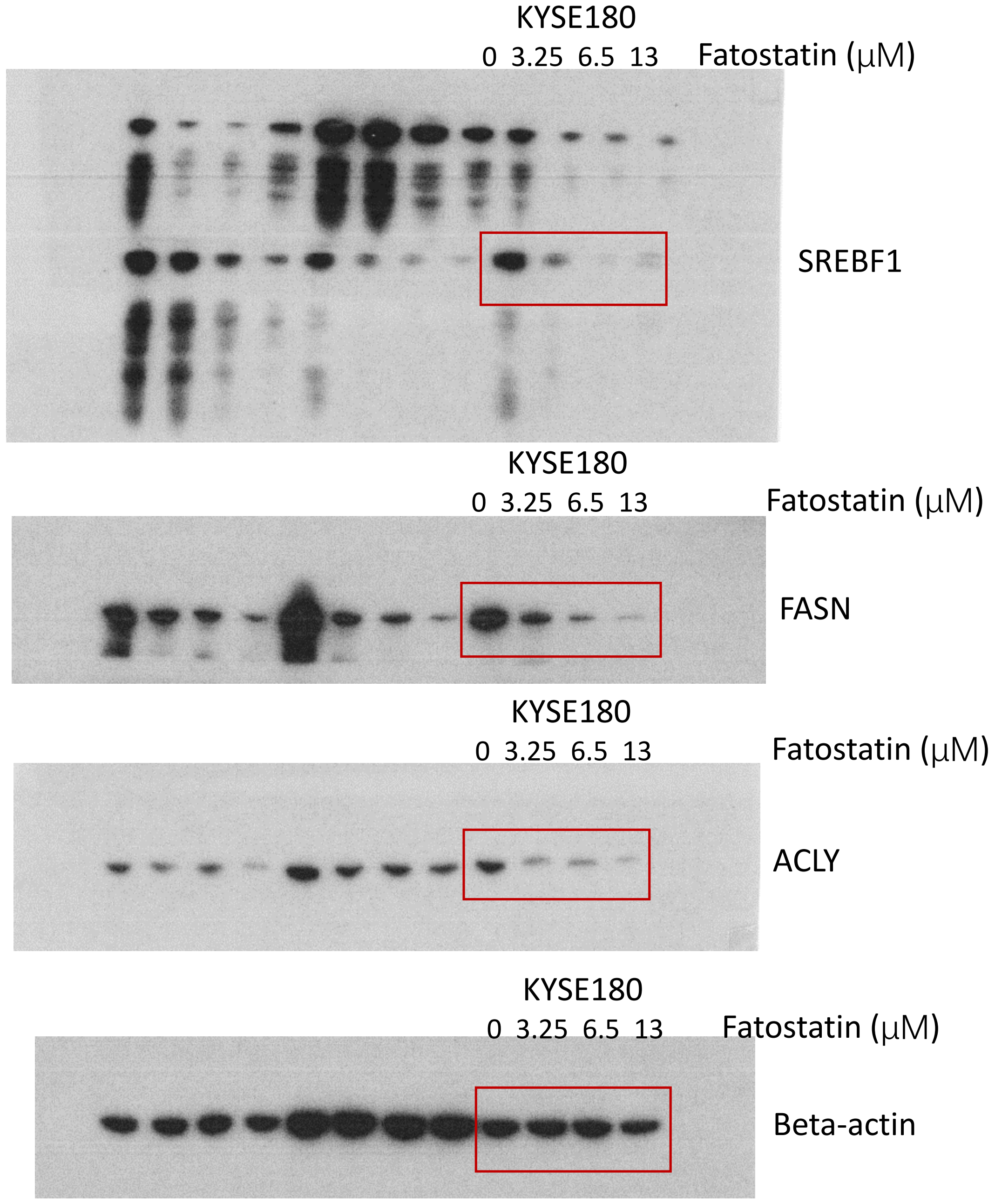

Supplement: Supplementary file 10 — Source Data [file 41467_2021_24656_MOESM10_ESM.zip › 5-Source Data-V0626/1-Source data for original gels and blots-V0626.pdf]
